# Review and analysis of the overlapping threats of carbapenem and polymyxin resistant *E. coli* and *Klebsiella* in Africa

**DOI:** 10.1186/s13756-023-01220-4

**Published:** 2023-04-04

**Authors:** Danielle M. Venne, David M. Hartley, Marissa D. Malchione, Michala Koch, Anjali Y. Britto, Jesse L. Goodman

**Affiliations:** 1 Center on Medical Product Access, Safety and Stewardship, Georgetown University, 3900 Reservoir Road, Washington, DC 20057 USA; 2 James M. Anderson Center for Health Systems Excellence, Cincinnati Children’s Hospital, 3333 Burnet Avenue, Cincinnati, OH 45229 USA; 3 Department of Pediatrics, College of Medicine, University of Cincinnati, Cincinnati, OH 45229 USA; 4 Sabin Vaccine Institute, Influenza Vaccine Innovation, 2175 K St NW, Washington, DC 20037 USA

## Abstract

**Background:**

Carbapenem-resistant Enterobacterales are among the most serious antimicrobial resistance (AMR) threats. Emerging resistance to polymyxins raises the specter of untreatable infections. These resistant organisms have spread globally but, as indicated in WHO reports, the surveillance needed to identify and track them is insufficient, particularly in less resourced countries. This study employs comprehensive search strategies with data extraction, meta-analysis and mapping to help address gaps in the understanding of the risks of carbapenem and polymyxin resistance in the nations of Africa.

**Methods:**

Three comprehensive Boolean searches were constructed and utilized to query scientific and medical databases as well as grey literature sources through the end of 2019. Search results were screened to exclude irrelevant results and remaining studies were examined for relevant information regarding carbapenem and/or polymyxin(s) susceptibility and/or resistance amongst *E. coli* and *Klebsiella* isolates from humans. Such data and study characteristics were extracted and coded, and the resulting data was analyzed and geographically mapped.

**Results:**

Our analysis yielded 1341 reports documenting carbapenem resistance in 40 of 54 nations. Resistance among *E. coli* was estimated as high (> 5%) in 3, moderate (1–5%) in 8 and low (< 1%) in 14 nations with at least 100 representative isolates from 2010 to 2019, while present in 9 others with insufficient isolates to support estimates. Carbapenem resistance was generally higher among *Klebsiella*: high in 10 nations, moderate in 6, low in 6, and present in 11 with insufficient isolates for estimates. While much less information was available concerning polymyxins, we found 341 reports from 33 of 54 nations, documenting resistance in 23. Resistance among *E. coli* was high in 2 nations, moderate in 1 and low in 6, while present in 10 with insufficient isolates for estimates. Among *Klebsiella*, resistance was low in 8 nations and present in 8 with insufficient isolates for estimates. The most widespread associated genotypes were, for carbapenems, *bla*_OXA-48,_ *bla*_NDM-1_ and *bla*_OXA-181_ and, for polymyxins, *mcr*-1, *mgrB*, and *phoPQ*/*pmrAB*. Overlapping carbapenem and polymyxin resistance was documented in 23 nations.

**Conclusions:**

While numerous data gaps remain, these data show that significant carbapenem resistance is widespread in Africa and polymyxin resistance is also widely distributed, indicating the need to support robust AMR surveillance, antimicrobial stewardship and infection control in a manner that also addresses broader animal and environmental health dimensions.

**Supplementary Information:**

The online version contains supplementary material available at 10.1186/s13756-023-01220-4.

## Introduction

Antimicrobial resistance (AMR) is of growing concern as multidrug resistant organisms (MDRO) become more prevalent globally, undermining the efficacy of medicines needed for the treatment of infections and threatening patient safety and economic wellbeing [1]. Carbapenem-resistant *Enterobacterales* (CRE) infections are of particular concern as treatment options are highly limited [2] with carbapenems considered critical drugs for treatment of infections with documented or suspected resistance to alternative antimicrobials. Healthcare environments are the dominant source of human exposure to MDRO such as CRE [3] but exposure may also occur in the community, where organisms spread not only after transfer from patients exposed in healthcare settings, but also through contact with food, animals, and the environment [4–8].

Resistance to carbapenems arises through intrinsic or acquired mechanisms [3]. Acquired resistance [9–14] typically occurs due to carbapenemase enzymes encoded on plasmids or other genetic elements that are readily transferred among organisms [2, 15]. Major resistance determinants present worldwide include expression of Class A *Klebsiella pneumoniae* carbapenemases (KPC), Class B metallo-β-lactamases such as New Delhi metallo-β-lactamases (NDM), Verona integron-encoded metallo-β-lactamases (VIM), Imipenemase metallo-β-lactamases (IMP), and Class D oxacillinase β-lactamases (OXA), and alterations in outer membrane proteins (OMP) [15]. The polymyxin antibiotics, including polymyxin E (colistin) and polymyxin B, hereon in referred to as polymyxin(s), are polycationic peptides widely used until the 1970s, when largely abandoned as less toxic antibiotics became available [16, 17]. Currently, as one of few antimicrobial classes effective against CRE, polymyxins have regained importance. Determinants of acquired polymyxin resistance include transferable plasmid encoded mobile colistin resistance (*mcr)* genes as well as chromosomally encoded genes such as *mgrB*, *phoP*/*phoQ*, and *pmrA*/*pmrB* [16, 18]. The risk of organisms acquiring both carbapenem and polymyxin resistance is alarming as it severely limits treatment options. While rare to date, such dual resistance has been increasingly documented [19–22].

Despite the association of MDRO with excess morbidity, mortality and costs, major gaps exist in surveillance, particularly in under-resourced areas [23]. The WHO Global Action Plan to Tackle AMR (GAP-AMR) provides a roadmap for the treatment and prevention of resistant infections [24]. Since 2014, WHO has encouraged collection of data on carbapenem susceptibility and has published the limited available data in reports of the Global Antimicrobial Resistance Use and Surveillance System (GLASS) [1, 25–27]. In 2018, noting that only 7 of 47 WHO Africa nations had reported data on CRE to WHO [12, 28–30], we developed search and metanalytic approaches to utilize data from diverse sources to estimate and map carbapenem resistance and related genotypes in the WHO Africa region. We were able to identify and analyze data from 31 of 47 nations [2] documenting carbapenem-resistant *Escherichia coli* or *Klebsiella* spp. in 22, typically at low to moderate levels [2]. We subsequently refined these approaches to characterize carbapenem and polymyxin resistance and their concerning overlaps in Southeast Asia [31].

Since our initial study, reporting on carbapenem resistance in Africa has increased [32–34] but comprehensive analyses are not available. Information on polymyxin resistance is more limited but recent reviews document *mcr* plasmids as causes of resistance in several African nations [35, 36]. The 2020 WHO GLASS report included only 10 of 54 nations reporting data on carbapenems and just 4 on polymyxins. Given these persistent data gaps there is a major unmet need for information to inform medical and public health investments, strategies and practices. We applied our previously-developed approaches to locate available useful data on polymyxin/colistin resistance and related genes, as well as to broadly update analyses of carbapenem resistance to reflect emerging data and extend the scope of study to all continental Africa. The results provide a comprehensive database and maps of carbapenem and polymyxin resistance in Africa, documenting the significant ongoing spread of both throughout the continent.

## Methods

### Literature review and other data sources

Three comprehensive Boolean searches were constructed and utilized to query scientific and medical databases (Embase, Global Health, PubMed and Web of Science). Grey literature sources including ProMED-mail [37], ResistanceMap [38] and HealthMap [39] were also examined for data from African nations, as described [2, 31]. Data were further supplemented by review and, where meeting criteria, extraction of relevant primary data located based on citations identified through included studies or from other referenced reviews and meta-analyses, as well as directly utilizing data from World Health Organization GLASS reports [1, 25–27] and author correspondence. As detailed previously, for nations with fewer than 4 reports from these sources, manual Google Scholar searches were conducted and additional sources such as African Journals Online, Bioline International and Global Index Medicus were hand-searched for relevant documents [2].

### Search strategy

As described [2, 31], search strategies were designed and executed to capture data describing susceptibility or resistance, and/or related genotypic findings, of *Escherichia coli* and *Klebsiella* isolates from humans. The searches (search operators capitalized) generally followed the structure of place (e.g. terms for Africa OR country names) AND terms for AMR (including general OR specific AMR terms OR synonym drug terms) AND species/mechanisms (including resistance enzymes and plasmid-mediated genotypes). As detailed (Additional file 1) the search strings also contained MeSH terms to optimize sensitivity while enhancing specificity. The first database search updated data from the WHO Africa Region nations (United Nations geoscheme) through 31 December 2019 [2]. The second search identified data published from 1 January 1996 to 31 December 2019 on carbapenem susceptibility or resistance for seven African countries not included in our original report (Djibouti, Egypt, Libya, Morocco, Tunisia, Somalia, and Sudan) [2]. The final search for 1 January 1996 to 31 December 2019 identified data for polymyxin susceptibility or resistance for all African nations.

### Exclusion and inclusion criteria and data collection

Two authors (DMV and AYB) screened search result titles and excluded irrelevant materials. Remaining studies were examined for relevant information regarding carbapenem and/or polymyxin(s) susceptibility and/or resistance amongst *E. coli* and *Klebsiella* isolates from humans. Minimum criteria for inclusion in the study database were description of study design and sampling process, characteristics of participants, places and dates of data collection and use of recognized, standardized testing methods at the time of performance. Studies not including these data elements were excluded. Data were extracted and coded from studies meeting criteria and any coding questions resolved through mutual agreement amongst researchers.

Underlying data from 313 reports in our previous dataset [2] on carbapenem resistance in WHO Africa nations (from searches through 31 June 2017) were also incorporated into the current dataset. If a newly found study reported data duplicative of or overlapping with that included in earlier analyses, only the original report was included. We also examined the results of database searches for similar reports (e.g. in terms of country, dates and species) to detect potentially duplicative or overlapping reporting of the same data. In circumstances where searches yielded duplicative or overlapping data, the most complete study was utilized unless both included unique data, in which case any additional details from the second report were included on a separate line of the database without duplicate reporting. When a study provided potentially important findings, but substantive uncertainties were present, authors were contacted, when possible, for clarification. Outreach to authors was made for 167 studies and responses obtained for 85, of which 55 were included in the manuscript (see acknowledgements).

### Database construction, definitions and data entry

A structured Microsoft Excel Version 1808 (Microsoft Corp., Redmond, WA, USA) template with predefined attributes was developed and utilized, as described [2, 31]. Data extracted included study characteristics, patient populations, and phenotypic and genotypic carbapenem and polymyxin resistance. Study type was classified as clinical laboratory-based, case series, outbreak, or surveillance, and populations were classified as from acute or chronic healthcare facilities, community-based, travelers or unknown [31]. Selected subpopulations, if studied, were defined by clinical attributes (e.g. pregnant, intensive care unit, clinical syndrome), travel status (e.g. immigrants, refugees) and/or occupation (e.g. farmers, students, healthcare workers). WHO age classification was utilized where applicable, unless age was otherwise classified by authors [31, 40].

Reports on subsets of laboratory isolates selected based on their resistance properties were coded noting the selection criteria utilized (e.g. ESBL or CRE). If results of susceptibility testing to multiple carbapenems were reported, all data were entered in the database with the value for the drug with the highest percentage resistance then used to represent overall carbapenem resistance, so long as the numbers of isolates tested for each drug were similar. On occasions where the differences in total numbers of isolates tested against different carbapenems were large (e.g. an order of magnitude), we used results from the drug with the most isolates tested to represent resistance. Isolates reported as having intermediate susceptibility were classified as resistant. For studies presenting disaggregated susceptibility results (e.g. by ESBL status), data were reaggregated to reflect resistance in the entire original set of isolates. Documentation of specific carbapenem or polymyxin resistance-associated genotypes was recorded whenever available. For quality control, all database entries were checked and confirmed by an additional reviewer.

### Data analyses

#### Defining the presence of resistance and/or specific resistance genotypes

Any report of at least one carbapenem and/or polymyxin-resistant *E. coli* or *Klebsiella* isolate, or an isolate with a resistance-associated genotype, signified the presence of resistance in that nation. This included findings of phenotypic resistance or resistance inducing genotypes in any isolate, whether from population-based studies or narrower studies of outbreaks, case series, highly selected subpopulations, or from studies of isolates themselves selected for known resistance to any antibiotic(s) including carbapenem and polymyxin.

#### Crude national resistance proportion estimates

Analysis was conducted using R version 3.5.2 (R Core Team, 2014). To estimate crude national resistance proportions, data from studies with a minimum number of isolates tested (20 for carbapenems and 10 for polymyxin, given the paucity of available data) that were deemed to originate from reasonably ‘generalizable’ populations (i.e. representative of individuals in overall healthcare populations), were aggregated and analyzed across studies. These estimates excluded any data from outbreaks or from studies reporting resistance in certain highly selected subpopulations (burn injury, oncology or transplantation) that typically have levels of resistance significantly greater than general acute-care populations. Similarly, data reporting resistance among organisms selected based on their known resistance to any antibiotic were not considered generalizable and therefore not included in resistance estimates.

To better reflect recent resistance, crude resistance proportions were calculated using data available on isolates collected from 2010 onward. If the total of generalizable *E. coli* or *Klebsiella* isolates tested for susceptibility to carbapenems or polymyxin(s) from 2010 onward was at least 100, we calculated that nation’s mean and, across qualifying studies, median resistance proportions using R v.3.5.2. For nations with at least 100 generalizable isolates of *E. coli* or *Klebsiella*, a crude estimated median resistance category was assigned consistent with prior studies [2, 31] as follows: not detected, low (< 1%), moderate (1–5%) or high (> 5%). If the total of generalizable isolates for a nation was less than 100, a category of either ‘Insufficient isolates – Resistance detected’ or ‘Insufficient isolates – Resistance not detected’ was assigned.

### Geocoding and mapping

ArcGIS Desktop 10.6 (ESRI, Redlands, CA, USA) was used to map median resistance proportions and genotypes at the national level. Sample origin was geocoded at facility level, or to the closest local administrative unit such as City or State/Province using Google Maps.

### Data sharing

The supplementary material, including search strings (Additional file 1) and outputs (Additional file 2), explanation of data elements extracted for analyses (Additional file 3), and all study data (Additional file 4) are available through Mendeley.

## Results

### Data characteristics

The searches yielded 8631 studies of which 1191 passed initial screening and 749 then met inclusion criteria. Three were in French, all others were in English. Because a given study may contain data on more than one organism, sets of isolates, or populations, the 749 study documents yielded a total of 1479 unique data reports together providing data on carbapenem and/or polymyxin resistance from 48 of 54 African countries. Three nations (Egypt, Nigeria and South Africa) accounted for 647 (43.7%) of all reports in the database. In contrast, no relevant reports were identified from 6 nations and nearly 30 nations each accounted for less than 1% of reports.

Selected general attributes of the data reports are displayed in Table 1. Six hundred and ninety-two (46.8%) reported on *E. coli*, while 787 (53.2%) were on *Klebsiella* spp. More than half of the data reports (67.5%) were from clinical laboratory-based studies, while 22.6% were from case series, 8.2% from surveillance and 2% from outbreaks. Aside from 34.6% of reports of multiple sample sources, most reports were of isolates from urine (23.3%) or blood (20.6%). Subject ages were reported as all (30%), adults (20.2%) and children (13.1%) or as unknown (34.2%). The majority (83.4%) of reports included isolates collected in acute healthcare settings, others included community-based settings (29.0%), chronic health-care facilities (0.5%), unknown healthcare settings (4.2%), travelers (1.0%) and unknown sources (1.7%).

**Table 1:** Key data attributes

Age group	Number (%)	Population type	Number (%)	Study type	Number (%)	Specimen type	Number (%)	Specimen type	Number (%)	Species	Number (%)
Adolescent	37 (2.5%)	Community	429 (29.0%)	Case series	334 (22.6%)	Ascitic fluid	3 (0.2%)	Pus	17 (1.2%)	*E. coli* ^a^	692 (46.8%)
Adult	299 (20.2%)	HC-acute	1234 (83.4%)	Clinical lab	998 (67.5%)	Aspirate	1 (0.1%)	Rectal	1 (0.1%)	*K.* spp. ^b^	787 (53.2%)
All	444 (30.0%)	HC-long	8 (0.5%)	Outbreak	29 (2.0%)	BAL	1 (0.1%)	Respiratory	6 (0.4%)		
Child	194 (13.1%)	HC-unknown	62 (4.2%)	Surveillance	121 (8.2%)	Bedsore	1 (0.1%)	Sperm	1 (0.1%)		
Elderly	16 (1.1%)	Travelers	15 (1.0%)			Blood	304 (20.6%)	Sputum	9 (0.6%)		
Infant	56 (3.8%)	Unknown	25 (1.7%)			Catheter	2 (0.1%)	Stool	184 (12.4%)		
Neonate	61 (4.1%)					Cervicovaginal	2 (0.1%)	Tissue	7 (0.5%)		
Unknown	506 (34.2%)					CSF	8 (0.5%)	Umbilical	1 (0.1%)		
						Ear	6 (0.4%)	Unknown	62 (4.2%)		
						Endocervical	2 (0.1%)	Urine	345 (23.3%)		
						Endotracheal	3 (0.2%)	Vaginal	7 (0.5%)		
						ETA	4 (0.3%)	Wound	69 (4.7%)		
						Gastric fluid	2 (0.1%)				
						Hand	3 (0.2%)				
						IV fluid	1 (0.1%)				
						Multiple	512 (34.6%)				
						Nasal	7 (0.5%)				
						Otitis media	2 (0.1%)				
						Peritoneal fluid	11 (0.7%)				
						Peritoneum	1 (0.1%)				

Numbers and % of 1479 unique data reports including the indicated subgroups. In some categories total is > 1479 as reports may contain multiple subgroups

^a^*Escherichia coli*

^b^*Klebsiella* spp.

### Carbapenem resistance: overview of data from all years

There were a total of 1341 data reports, derived from 708 studies, providing data on carbapenem susceptibility from 48 of 54 nations (Table 2). These included 622 (46.4%) on *E. coli* isolates and 719 (53.6%) on *Klebsiella* from 48 and 42 nations, respectively. Of the total 1341 reports, 879 (65.5%) were from nations in WHO Africa (including 313 incorporated from the earlier analysis [2]) while 462 (34.5%) were from the other African nations. Phenotypic and or genotypic carbapenem resistance was reported among either species in 40 of 48 nations (83.3%) from which data were available. Specifically, resistance was detected among *E. coli* in 36 of 48 nations (75%) with available data and among *Klebsiella* in 35 of 42 (83.3%). There were no data available on *E. coli* or *Klebsiella* from 6 nations (Burundi, Comoros, Lesotho, Liberia, Seychelles and Swaziland) while data were available on *E. coli* but not *Klebsiella* from an additional 6 (Cape Verde, Djibouti, Eritrea, Guinea, Lesotho, Somalia and South Sudan). Tables 3 and 4 present national-level carbapenem resistance data for all years studied, including whether resistance was reported, specific genotypes detected and, for samples from generalizable studies, percent mean resistance.

**Table 2:** Available reports on *E. coli* and *Klebsiella* carbapenem and polymyxin susceptibility, resistance, and related genes

Nation	All reports on named species (reports identifying resistance or determinants related to resistance)				References
	Carbapenem		Polymyxin (colistin and polymyxin B)		
	*E. coli*	*Klebsiella*	*E. coli*	*Klebsiella*	
Algeria	33 (12)	37(18)	17(4)	22(3)	[48–97]
Angola	2(2)	2(2)	1(0)	1(0)	[98, 99]
Benin	6(4)	2(1)	2(0)	2(0)	[100–105]
Botswana	1(0)	1(0)	0	0	[106]
Burkina Faso	12(4)	13(0)	3(2)	1(0)	[25, 107–121]
Cameroon	11(3)	7(3)	3(2)	1(0)	[116, 122–133]
Cape Verde	1(0)	0	0	0	[116]
Central African Republic	2(0)	3(0)	0	0	[25, 134, 135]
Chad	8(4)	4(1)	1(0)	0	[57, 116, 136–141]
Congo	2 (0)	1 (1)	1(0)	0	[142, 143]
Cote d'lvoire	4(1)	5(1)	1 (1)	0	[144–150]
Democratic Republic of the Congo	3(0)	2(1)	0	0	[151–153]
Djibouti	2(1)	0	1(0)	0	[57, 154]
Egypt	106(66)	125 (98)	28 (14)	34(15)	[1, 25–27, 57, 78, 116, 155–293]
Equatorial Guinea	1(0)	1 (1)	0	0	[294]
Eritrea	1(0)	0	1(0)	0	[295]
Ethiopia	19(10)	27(17)	4(4)	6(4)	[1, 112, 296–316]
Gabon	4(0)	5(1)	0	0	[317–321]
Gambia	1 (1)	1 (1)	0	0	[322]
Ghana	15(5)	15(8)	1 (1)	1 (1)	[116, 323–337]
Guinea	1(0)	0	0	0	[116]
Guinea-Bissau	1(0)	1(0)	0	0	[338]
Kenva	26(11)	25 (21)	2(1)	2(0)	[116, 168, 268, 282, 339–362]
Libya	17(10)	22(20)	3(0)	8(4)	[57, 78, 363–385]
Madagascar	14(3)	12(6)	0	0	[1, 26, 27, 112, 116, 168, 386–395]
Malawi	4(3)	7(5)	1(0)	2(0)	[26, 27, 396–398]
Mali	4(3)	2(1)	1(0)	0	[1, 57, 399, 400]
Mauritania	1 (1)	1(0)	1 (1)	1 (1)	[401]
Mauritius	4(2)	6(5)	1(0)	2(1)	[25, 57, 168, 183, 402–404]
Morocco	24(13)	39(24)	6(2)	10(2)	[25, 57, 78, 116, 183, 268, 282, 405–433]
Mozambique	6(1)	4(0)	1(0)	0	[1, 116, 427, 434–438]
Namibia	1(0)	5(1)	0	0	[25, 183, 439]
Niger	5(2)	2(0)	1 (1)	1 (1)	[57, 440–443]
Nigeria	82(53)	80(46)	3203)	28(15)	[1, 27, 116, 444–561]
Rwanda	7(3)	6(2)	1 (1)	1 (1)	[562–568]
Sao Tome and Principe	1 (1)	1 (1)	1 (1)	0	[569]
Senegal	10(2)	11(6)	1 (1)	3(1)	[56, 78, 112, 116, 145, 570–581]
Sierra Leone	5(3)	4(3)	0	0	[116, 582, 583]
Somalia	1(0)	0	1(0)	0	[295]
South Africa	69(25)	109(82)	18(8)	20(10)	[1, 25, 78, 168, 183, 268, 282, 519, 584–663]
South Sudan	1(0)	0	0	0	[664]
Sudan	12(7)	10(5)	1 (1)	1 (1)	[1, 27, 112, 116, 665–673]
Tanzania	29(6)	26(7)	2(1)	2(1)	[56, 112, 116, 168, 674–700]
Togo	6(4)	4(3)	3(1)	3(1)	[116, 701–706]
Tunisia	34(14)	70(43)	15(4)	29(13)	[1, 26, 27, 78, 168, 183, 268, 282, 707–774]
Uganda	17(10)	16(11)	2(1)	2(1)	[1, 26, 27, 168, 775–788]
Zambia	3(1)	4(4)	0	0	[26, 27, 789, 790]
Zimbabwe	3 (1)	1 (1)	0	0	[116, 791, 792]
All reporting nations	942(622)	451 (19)	75(158)	76(183)	

Reports on carbapenem or polymyxin susceptibility were not identified from the following searched nations: Burundi, Comoros, Lesotho, Liberia, Seychelles and Swaziland

**Table 3:** Carbapenem resistance (R) and resistance determinants in *Escherichia coli* isolates: data from all years

				Findings in reports from all study years meeting criteria for generalizability					Identified resistance determinants
Nations	Number of reports	Specimens in all reports	Any R	Reports meeting criteria	Total specimens meeting criteria	Range of specimens among studies	Resistant specimens (#)	Resistant specimens (%)	
Algeria	33	4304	Y	15	4201	30—1184	13	0.3	NDM-5, OXA-48, OXA-181, VIM-19
Angola	2	52	Y	0	0	–	–	–	NDM-1, NDM-5, OXA-181
Benin	6	692	Y	5	687	84–221	18	2.6	
Botswana	1	27	N	0	0	–	–	–	
Burkina Faso	12	787	Y	6	743	26–296	5	0.7	GES, OXA, OXA-181
Cameroon	11	330	Y	6	313	21–163	7	2.2	NDM-4
Cape Verde	1	1	N	0	0	–	–	–	
CAR	2	84	N	2	84	33–51	0	0	
Chad	8	402	Y	5	382	31–128	6	1.6	NDM-5, OXA, OXA-181
Congo	2	112	Y	2	112	23–89	4	3.6	OXA-48
Côte d'Ivoire	4	145	Y	2	121	57–64	0	0	
DRC	3	451	N	3	451	21–376	0	0	
Djibouti	2	32	Y	1	31	–	0	0	OXA-48
Egypt	106	8657	Y	56	7549	20–3177	425	5.6	KPC, GES, IMP, NDM, NDM-1, NDM-5, OXA-48, OXA-181, VIM, VIM-1, VIM-2
Equatorial Guinea	1	39	N	1	39	–	0	0	
Eritrea	1	14	N	0	0	–	–	–	
Ethiopia	19	1794	Y	12	1729	31–235	54	3.1	KPC
Gabon	4	142	N	3	133	30–57	0	0	
Gambia	1	8	Y	0	0	–	–	–	
Ghana	15	621	Y	9	568	25–124	27	4.8	NDM-1, OXA-48
Guinea	1	1	N	0	0	–	–	–	
Guinea-Bissau	1	83	N	1	83	–	0	0	
Kenya	26	10,654	Y	18	10,554	25–5165	57	0.5	
Libya	17	1387	Y	8	1154	75–346	62	5.4	OXA-48
Madagascar	14	1381	Y	8	1355	31–672	7	0.5	
Malawi	4	2601	Y	2	2592	657–1935	54	2.1	NDM-5, OXA-48
Mali	4	211	Y	3	210	31–132	25	11.9	NDM-4, OXA-181
Mauritania	1	366	Y	1	366	–	4	1.1	
Mauritius	4	202	Y	1	183	–	5	2.7	OXA-181
Morocco	24	3585	Y	10	3459	49–1174	41	1.2	IMP-1, OXA-48
Mozambique	6	188	Y	3	161	35–75	0	0	
Namibia	1	23	N	1	23	–	0	0	
Niger	5	720	Y	3	502	27–434	0	0	OXA-181
Nigeria	82	5072	Y	43	4161	21–400	319	7.7	GES, NDM, OXA, OXA-48, OXA-181, VIM
Rwanda	7	3009	Y	6	3002	55–2473	201	6.7	
Sao Tome and Principe	1	30	Y	0	0	–	–	–	OXA-181
Senegal	10	581	Y	4	554	33–398	1	0.2	OXA-48
Sierra Leone	5	14	Y	0	0	–	–	–	DIM-1, OXA-58, VIM
Somalia	1	27	N	1	27	–	0	0	
South Africa	69	36,224	Y	41	35,930	20–14,348	333	0.9	NDM, NDM-1, NDM-5, OXA-48, VIM, VIM-1
South Sudan	1	65	N	0	0	–	–	–	
Sudan	12	1085	Y	4	978	71–458	72	7.4	IMP, NDM
Tanzania	29	1977	Y	16	1793	20–837	18	1	KPC, IMP, NDM, OXA-48, VIM
Togo	6	238	Y	2	109	35–74	1	0.9	
Tunisia	34	23,696	Y	17	23,619	31–9485	214	0.9	KPC-2, NDM-1, OXA-48
Uganda	17	1532	Y	6	1302	22–930	167	12.8	KPC, IMP, OXA-48, VIM
Zambia	3	477	Y	3	477	56–343	341	71.5	
Zimbabwe	3	204	Y	2	203	23–180	27	13.3	
All reporting countries	622	114,327	Y	332	109,940	20–14,348	2508	2.3**	

*Y* One or more resistant isolates identified phenotypically or genotypically

*N* No resistant isolates identified phenotypically or genotypically

^**^Calculation should not be considered an estimate of overall resistance due to varying totals of specimens meeting criteria across nations

–Data not available

**Table 4:** Carbapenem resistance (R) and resistance determinants in *Klebsiella* spp. isolates: data from all years

				Findings in reports from all study years meeting criteria for generalizability					Identified resistance determinants
Nations	Number of reports	Specimens in all reports	Any R	Reports meeting criteria	Total specimens meeting criteria	Range of specimens among studies	Resistant specimens (#)	Resistant specimens (%)	
Algeria	37	2174	Y	12	1968	24–608	25	1.3	KPC-3, NDM, NDM-1, OXA-48, VIM-19
Angola	2	49	Y	0	0	–	–	–	NDM-1, NDM-5, OXA-181
Benin	2	51	Y	1	41	–	1	2.4	
Botswana	1	40	N	0	0	–	–	–	
Burkina Faso	13	297	N	4	242	20–109	0	0	
Cameroon	7	299	Y	4	276	28–99	5	1.8	
CAR	3	77	N	2	67	24–43	0	0	
Chad	4	87	Y	3	86	23–35	1	1.2	OXA
Congo	1	12	Y	0	0	–	–	–	
Côte d'Ivoire	5	237	Y	4	229	22–107	0	0	
DRC	2	167	Y	2	167	21–146	1	0.6	
Egypt	125	7320	Y	59	5501	20–594	1545	28.1	KPC, KPC-2, IMP, IMP-1, NDM, NDM-1, OXA-48, VIM, VIM-1, VIM-2
Equatorial Guinea	1	30	Y	1	30	–	1	3.3	
Ethiopia	27	808	Y	9	675	30–154	78	11.6	KPC, NDM-1
Gabon	5	161	Y	2	146	67–79	0	0	NDM-7
Gambia	1	9	Y	0	0	–	–	–	
Ghana	15	537	Y	10	505	20–107	85	16.8	NDM, OXA-48
Guinea-Bissau	1	91	N	1	91	–	0	0	
Kenya	25	1471	Y	15	1419	25–272	131	9.2	KPC, NDM, NDM-1, NDM-5, OXA-48, OXA-58, VIM
Libya	22	709	Y	8	514	24–158	202	39.3	KPC, NDM, NDM-1, OXA-48
Madagascar	12	472	Y	6	418	22–122	13	3.1	NDM-1
Malawi	7	1315	Y	2	1276	173–1103	60	4.7	KPC-2, OXA-48
Mali	2	67	Y	2	67	26–41	7	10.4	
Mauritania	1	137	N	1	137	–	0	0	
Mauritius	6	235	Y	2	222	104–118	13	5.9	NDM-1, OXA-181
Morocco	39	1784	Y	10	1380	24–389	69	5	IMP-1, NDM-1, OXA-48, VIM-1
Mozambique	4	63	N	1	21	–	0	0	
Namibia	5	313	Y	2	303	23–280	1	0.3	
Niger	2	21	N	0	0	–	–	–	
Nigeria	80	4111	Y	42	3524	21–600	318	9	GES, NDM, NDM-1, NDM-5, OXA, OXA-48, OXA-181, VIM
Rwanda	6	1222	Y	5	1214	22–975	108	8.9	
Sao Tome and Principe	1	4	Y	0	0	–	–	–	OXA-181
Senegal	11	249	Y	5	173	21–40	2	1.2	OXA-48
Sierra Leone	4	15	Y	0	0	–	–	–	DIM-1, OXA-58, VIM
South Africa	109	45,588	Y	53	42,915	20–15,589	4214	9.8	KPC, KPC-2, GES, IMP, NDM, NDM-1, OMP, OXA, OXA-48, OXA-181, OXA-232, VIM, VIM-1
Sudan	10	988	Y	5	940	21–404	98	10.4	IMP, NDM
Tanzania	26	947	Y	14	790	20–139	16	2	KPC, IMP, NDM, OXA-48, VIM
Togo	4	165	Y	1	86	–	3	3.5	OXA-181
Tunisia	70	12,842	Y	26	12,117	21–2826	1417	11.7	KPC, NDM, NDM-1, OMP, OXA-48, OXA-58, OXA-232, VIM, VIM-4
Uganda	16	319	Y	3	116	22–55	14	12.1	KPC, IMP, NDM-1, OXA-48, VIM
Zambia	4	683	Y	4	683	58–432	435	63.7	
Zimbabwe	1	130	Y	1	130	–	10	7.7	
All reporting countries	719	86,296	Y	322	78,469	20–15,589	8873	11.3**	

*Y* One or more resistant isolates identified phenotypically or genotypically

*N* No resistant isolates identified phenotypically or genotypically

**Calculation should not be considered an estimate of overall resistance due to varying totals of specimens meeting criteria across nations

–Data not available

#### Carbapenem resistance among more recent E. coli isolates

**Fig. 1:**
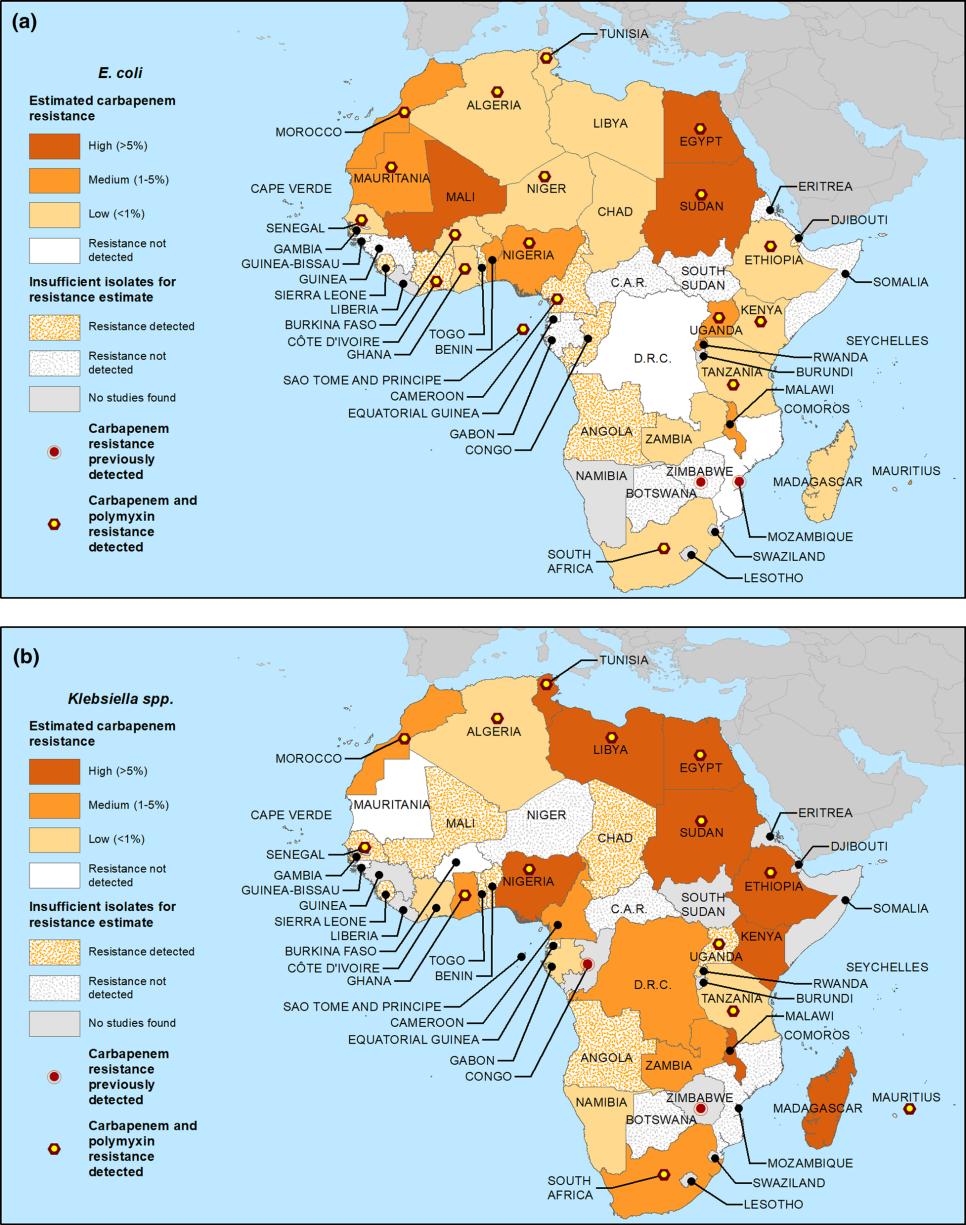
Estimated crude median national carbapenem resistance proportions for **a** *E. coli* and **b** *Klebsiella* spp. for studies including samples from 2010 and later. For those nations with ≥ 100 isolates from qualifying studies (see Methods), median proportions across studies were calculated. Where < 100 isolates, data were deemed insufficient to estimate proportions and resistance is represented as either detected or not

Table 5 displays carbapenem resistance data for *E. coli* based on samples collected in 2010 and later, including the mean and range of resistance percentages across studies, and, for nations with at least 100 generalizable isolates since 2010, crude estimated national resistance proportions (median across qualifying reports). Three nations (Egypt, Mali and Sudan) had high estimated resistance. Eight (Benin, Malawi, Mauritania, Mauritius, Morocco, Nigeria, Rwanda and Uganda) had moderate estimated resistance, and resistance in 14 nations (Algeria, Burkina Faso, Chad, Ethiopia, Ghana, Kenya, Libya, Madagascar, Niger, Senegal, South Africa, Tanzania, Tunisia and Zambia) was estimated as low. Resistance was not detected among ≥ 100 *E. coli* isolates from either the Democratic Republic of the Congo or Mozambique. Among nations with insufficient *E. coli* isolates to allow estimates, resistance was detected in nine (Angola, Cameroon, Congo, Côte d’Ivoire, Djibouti, Gambia, Sao Tome and Principe, Sierra Leone and Togo) and not detected in 11 (Botswana, Cape Verde, Central African Republic, Equatorial Guinea, Eritrea, Gabon, Guinea, Guinea-Bissau, Somalia, South Sudan and Zimbabwe). No relevant data were identified from Namibia. Resistance data for *E. coli* are mapped in Fig. 1a.

**Table 5:** Carbapenem resistance (R) estimates and data for *Escherichia coli* isolates from studies including samples from 2010 and later

				Findings in reports from all study years meeting criteria for generalizability							Resistance estimate category
Nations	Number of reports	Specimens in all reports	Any R	Reports meeting criteria	Total specimens meeting criteria	Range of specimens among studies	Resistant specimens (#)	Resistant specimens (%)	Resistant range (%)	Median R	
Algeria	21	2434	Y	10	2371	30–1184	13	0.5	0–12.7	0	Low
Angola	2	52	Y	0	0	-	-	-	-	-	Insufficient isolates—resistance detected
Benin	5	503	Y	4	498	84–221	11	2.2	0–8	2.3	Moderate
Botswana	1	27	N	0	0	-	-	-	-	-	Insufficient isolates—resistance not detected
Burkina Faso	10	651	Y	5	611	26–296	5	0.8	0–16.1	0	Low
Cameroon	6	69	Y	2	54	24–30	5	9.3	0–16.7	N/A*	Insufficient isolates—resistance detected
Cape Verde	1	1	N	0	0	-	-	-	-	-	Insufficient isolates—resistance not detected
CAR	2	84	N	2	84	33–51	0	0	0–0	N/A*	Insufficient isolates—resistance not detected
Chad	8	402	Y	5	382	31–128	6	1.6	0–4.7	0	Low
Congo	1	89	Y	1	89	-	3	3.4	-	N/A*	Insufficient isolates—resistance detected
Côte d'Ivoire	2	71	Y	1	57	-	0	0	-	N/A*	Insufficient isolates—resistance detected
DRC	3	451	N	3	451	21–376	0	0	0–0	0	Resistance not detected
Djibouti	2	32	Y	1	31	-	0	0	-	N/A*	Insufficient isolates—resistance detected ^
Egypt	71	4094	Y	36	3274	21–486	377	11.5	0–83.3	7.9	High
Equatorial Guinea	1	39	N	1	39	-	0	0	-	N/A*	Insufficient isolates—resistance not detected
Eritrea	1	14	N	0	0	-	-	-	-	-	Insufficient isolates—Resistance not detected
Ethiopia	19	1794	Y	12	1729	31–235	54	3.1	0–41.8	0.9	Low
Gabon	3	85	N	2	76	30–46	0	0	0–0	N/A*	Insufficient isolates—RESISTANCE not detected
Gambia	1	8	Y	0	0	-	-	-	-	-	Insufficient isolates—resistance detected
Ghana	12	394	Y	6	341	25–118	27	7.9	0–40.6	0	Low
Guinea	1	1	N	0	0	-	-	-	-	-	Insufficient isolates—resistance not detected
Guinea-Bissau	1	83	N	1	83	-	0	0	-	N/A*	Insufficient isolates—resistance not detected
Kenya	13	8603	Y	11	8595	25–5165	37	0.4	0–13	0	Low
Libya	14	1133	Y	7	1035	75–346	62	6	0–52	0.6	Low
Madagascar	9	1190	Y	5	1171	46–672	7	0.6	0–2	0.6	Low
Malawi	3	2600	Y	2	2592	657–1935	54	2.1	1.4–4.1	2.7	Moderate
Mali	3	164	Y	2	163	31–132	25	15.3	3.3–18.2	10.7	High
Mauritania	1	366	Y	1	366	-	4	1.1	-	1	Moderate
Mauritius	2	184	Y	1	183	-	5	2.7	-	3	Moderate
Morocco	15	3292	Y	7	3197	83–1174	41	1.3	0–5.7	1.1	Moderate
Mozambique	5	176	N	3	161	35–75	0	0	0–0	0	Resistance not detected
Niger	4	679	Y	2	461	27–434	0	0	0–0	0	Low
Nigeria	62	3095	Y	30	2567	21–278	265	10.3	0–63	2.7	Moderate
Rwanda	5	417	Y	4	410	55–139	8	2	0–8	1.7	Moderate
Sao Tome and Principe	1	30	Y	0	0	-	-	-	-	-	Insufficient isolates—resistance detected
Senegal	6	174	Y	3	156	33–74	1	0.6	0–3	0	Low
Sierra Leone	5	14	Y	0	0	-	-	-	-	-	Insufficient isolates—resistance detected ^
Somalia	1	27	N	1	27	-	0	0	-	N/A*	Insufficient isolates—resistance not detected
South Africa	36	24,270	Y	22	24,135	20–14,348	264	1.1	0–82.6	0	Low
South Sudan	1	65	N	0	0	-	-	-	-	-	Insufficient isolates—Resistance NOT detected
Sudan	10	614	Y	3	520	71–326	72	13.8	9–36.6	10.7	High
Tanzania	22	912	Y	13	819	20–164	18	2.2	0–19.2	0	Low
Togo	5	164	Y	1	35	-	0	0	-	N/A*	Insufficient isolates—resistance detected
Tunisia	17	21,324	Y	10	21,299	48–9485	207	1	0–3.7	0.6	Low
Uganda	13	598	Y	5	372	22–181	18	4.8	0–19	4.5	Moderate
Zambia	3	477	Y	3	477	56–343	341	71.5	0–99.3	0	Low
Zimbabwe	2	24	N	1	23	-	0	0	-	N/A*	Insufficient isolates—resistance not detected
All reporting countries	432	81,970	Y	229	78,934	20–14,348	1930	2.4**	–	–	–

*Y* one or more resistant isolates identified phenotypically or genotypically

*N* no resistant isolates identified phenotypically or genotypically

^Only genotypic resistance reported

*Insufficient isolates (< 100) for carbapenem resistance estimate

**Calculation should not be considered an estimate of overall resistance due to varying totals of specimens meeting criteria across nations

-Data not available

–Not calculated

#### Carbapenem resistance among more recent *Klebsiella* isolates

Median carbapenem resistance among recent *Klebsiella* isolates (Table 6) was estimated as high in 10 nations (Egypt, Ethiopia, Kenya, Libya, Madagascar, Malawi, Mauritius, Nigeria, Sudan and Tunisia). Six nations had moderate estimated resistance (Cameroon, Democratic Republic of the Congo, Ghana, Morocco, South Africa and Zambia), while resistance in 6 others (Algeria, Côte d’Ivoire, Gabon, Namibia, Rwanda and Tanzania) was estimated as low. Burkina and Mauritania had no resistance detected in ≥ 100 isolates. Among nations with insufficient *Klebsiella* isolates to allow estimates, resistance was detected in 11 (Angola, Benin, Chad, Equatorial Guinea, Gambia, Mali, Sao Tome and Principe, Senegal, Sierra Leone, Togo and Uganda) and not detected in 5 (Botswana, Central African Republic, Guinea-Bissau, Mozambique and Niger). No relevant data were identified from 8 nations (Cape Verde, Congo, Djibouti, Eritrea, Guinea, Somalia, South Sudan and Zimbabwe). Resistance data for *Klebsiella* are mapped in Fig. 1b.

**Table 6:** Carbapenem resistance (R) estimates and data for *Klebsiella* spp. isolates from studies including samples from 2010 and later

				Findings in reports from all study years meeting criteria for generalizability							Resistance estimate category
Nations	Number of reports	Specimens in all reports	Any R	Reports meeting criteria	Total specimens meeting criteria	Range of specimens among studies	Resistant specimens (#)	Resistant specimens (%)	Resistant range (%)	Median R	
Algeria	24	1205	Y	6	1029	24–608	25	2.4	0–20	0	Low
Angola	2	49	Y	0	0	-	-	-	-	-	Insufficient isolates—resistance detected
Benin	2	51	Y	1	41	-	1	2.4	-	N/A*	Insufficient isolates—Resistance detected
Botswana	1	40	N	0	0	-	-	-	-	-	Insufficient isolates—resistance not detected
Burkina Faso	11	234	N	2	179	70–109	0	0	0–0	0	Resistance not detected
Cameroon	3	154	Y	2	151	52–99	4	2.6	0–4	2	Moderate
CAR	2	34	N	1	24	-	0	0	-	N/A*	Insufficient isolates—Resistance not detected
Chad	4	87	Y	3	86	23–35	1	1.2	0–2.9	N/A*	Insufficient isolates—resistance detected
Côte d'Ivoire	2	115	Y	1	107	-	0	0	-	0	Low
DRC	2	167	Y	2	167	21–146	1	0.6	0–4.8	2.4	Moderate
Egypt	94	4925	Y	45	3617	20–425	1321	36.5	0–86.4	26	High
Equatorial Guinea	1	30	Y	1	30	-	1	3.3	-	N/A*	Insufficient isolates—resistance detected
Ethiopia	27	808	Y	9	675	30–154	78	11.6	0–30	10.7	High
Gabon	5	161	Y	2	146	67–79	0	0	0–0	0	Low
Gambia	1	9	Y	0	0	-	-	-	-	-	Insufficient isolates—Resistance detected
Ghana	12	366	Y	7	334	20–91	84	25.1	0–57.1	1.6	Moderate
Guinea-Bissau	1	91	N	1	91	-	0	0	-	N/A*	Insufficient isolates—resistance not detected
Kenya	17	964	Y	10	929	25–272	117	12.6	0–30	5.5	High
Libya	20	655	Y	7	464	24–158	202	43.5	0–92	26.9	High
Madagascar	8	306	Y	4	261	22–122	13	5	0–17	8.6	High
Malawi	5	1310	Y	2	1276	173–1103	60	4.7	2.7–17.3	10	High
Mali	2	67	Y	2	67	26–41	7	10.4	0–17.1	N/A*	Insufficient isolates—resistance detected
Mauritania	1	137	N	1	137	-	0	0	-	0	Resistance not detected
Mauritius	3	223	Y	2	222	104–118	13	5.9	1.9–9	5.4	High
Morocco	27	1671	Y	9	1348	24–389	69	5.1	0–22.5	3.1	Moderate
Mozambique	3	44	N	1	21	-	0	0	-	N/A*	Insufficient isolates—resistance not detected
Namibia	1	280	Y	1	280	-	1	0.4	-	0.4	Low
Niger	1	9	N	0	0	-	-	-	-	-	Insufficient isolates—resistance not detected
Nigeria	58	2642	Y	28	2343	21–600	287	12.2	0–81	8.9	High
Rwanda	5	247	Y	4	239	22–91	4	1.7	0–4.6	0	Low
Sao Tome and Principe	1	4	Y	0	0	-	-	-	-	-	Insufficient isolates—resistance detected
Senegal	5	116	Y	2	55	21–34	2	3.6	2.9–5	N/A*	Insufficient isolates—resistance detected
Sierra Leone	4	15	Y	0	0	-	-	-	-	-	Insufficient isolates—resistance detected ^
South Africa	62	37,049	Y	27	34,593	21–15,589	4051	11.7	0–90.1	3.5	Moderate
Sudan	8	576	Y	4	536	21–249	98	18.3	0–58	14.3	High
Tanzania	19	689	Y	11	618	20–139	16	2.6	0–13.6	0	Low
Togo	3	79	Y	0	0	-	-	-	-	-	Insufficient isolates—resistance detected
Tunisia	38	10,256	Y	12	9766	24–2826	1414	14.5	0–41.2	12.9	High
Uganda	14	262	Y	2	61	22–39	1	1.6	0–4.3	N/A*	Insufficient isolates—resistance detected
Zambia	4	683	Y	4	683	58–432	435	63.7	1–99.2	4.3	Moderate
All reporting countries	503	66,810	Y	216	60,576	20–15,589	8306	13.7**	–	–	–

*Y* one or more resistant isolates identified phenotypically or genotypically

*N* no resistant isolates identified phenotypically or genotypically

^Only genotypic resistance reported

*Insufficient isolates (< 100) for carbapenem resistance estimate

**Calculation should not be considered an estimate of overall resistance due to varying totals of specimens meeting criteria across nations

-Data not available

–Not calculated

#### Carbapenem resistance genotypes

**Fig. 2:**
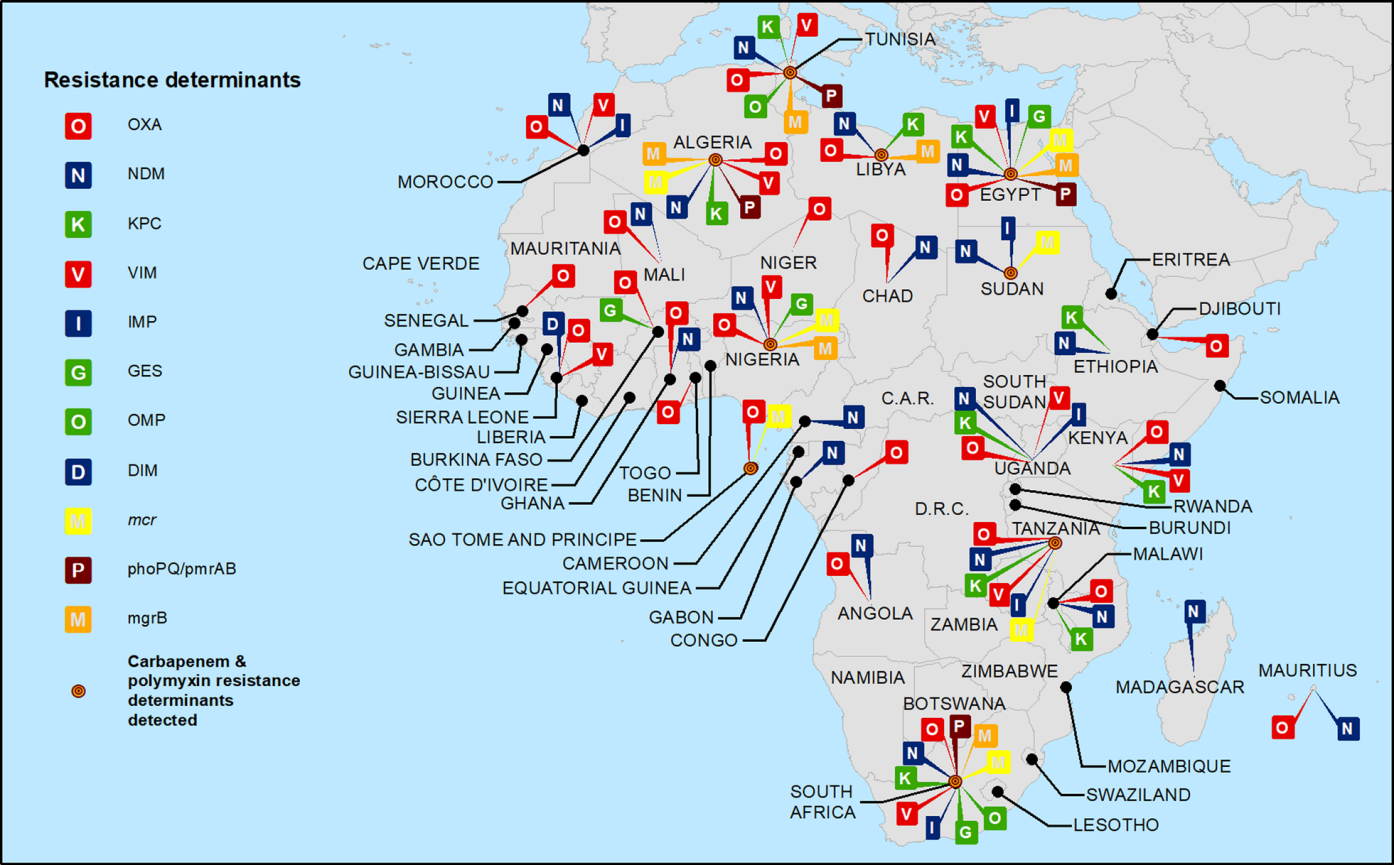
Carbapenem and polymyxin(s) resistance determinants reported from African nations

There were 94 data reports from 25 nations identifying at least one carbapenem resistance associated genotype among *E. coli* isolates (Table 3 and Fig. 2). The most common were *bla*_OXA-48_ and *bla*_OXA-181_, detected in 14 and 10 nations respectively. *bla*_VIM_ was identified in 6 nations and *bla*_NDM_, *bla*_NDM-1_ and *bla*_NDM-5_ each reported in 5. *bla*_GES_ was identified in 3 nations and *bla*_NDM-4_, *bla*_OXA_, and *bla*_VIM-1_ each identified in 2. *bla*_DIM-1_, *bla*_IMP_, *bla*_IMP-1_, *bla*_KPC_, *bla*_KPC-2_, *bla*_OXA-58,_ *bla*_VIM-2_ and *bla*_VIM-19_ were each noted in one nation.

For *Klebsiella* spp., there were 187 reports from 24 nations identifying at least one carbapenem resistance genotype (Table 4 and Fig. 2). As also noted for *E. coli*, *bla*_OXA-48_ and *bla*_OXA-181_ were most common, detected in 14 and 10 nations, respectively. *bla*_KPC_ was identified in 8 nations, *bla*_NDM-5_ and *bla*_VIM_ in 6, with *bla*_IMP_, *bla*_NDM_ and *bla*_NDM-1_ each found in 5. *bla*_KPC-2_ was identified in 3 nations and *bla*_IMP-1_, *bla*_NDM-4_, *bla*_OXA_ and *bla*_VIM-1_ were each identified in 2. *bla*_DIM-1_, *bla*_GES_, *bla*_KPC-3_, *bla*_VIM-2_ and *bla*_VIM-19_ were each identified in 1 nation.

### Polymyxin resistance: overview of data from all years

We found 341 unique data reports, derived from 208 studies, reporting data on polymyxin susceptibility from 33 of 54 African nations (Table 2). These reports included 158 (46.3%) on *E. coli* and 183 (53.7%) on *Klebsiella*, originating from 33 and 24 nations, respectively. Resistance was phenotypically or genotypically identified in 23 of the 33 nations (69.6%) from which any data were available. Tables 7 and 8 present national-level polymyxin resistance data for all years studied, including whether resistance was reported, specific genotypes detected and, for samples from generalizable studies, percent mean resistance.

**Table 7:** Polymyxin (colistin and polymyxin B) resistance (R) and resistance determinants in *Escherichia coli* isolates: data from all years

				Findings in reports from all study years meeting criteria for generalizability					Identified resistance determinants
Nations	Number of reports	Specimens in all reports	Any R	Reports meeting criteria	Total specimens meeting criteria	Range of specimens among studies	Resistant specimens (#)	Resistant specimens (%)	
Algeria	17	2249	Y	11	2235	13–1184	1	< 0.1	*mcr-1*
Angola	1	23	N	0	0	–	–	–	
Benin	2	97	N	1	92	–	0	0	
Burkina Faso	3	262	Y	3	262	26–205	40	15.3	
Cameroon	3	41	Y	1	30	–	30	100	
Chad	1	18	N	1	18	–	0	0	
Congo	1	89	N	1	89	–	0	0	
Côte d'Ivoire	1	177	Y	1	177	–	14	7.9	
Djibouti	1	31	N	1	31	–	0	0	
Egypt	28	1276	Y	14	678	11–212	32	4.7	*mcr*-*1*, mgrB, phoPQ/pmrAB
Eritrea	1	14	N	0	0	–	–	–	
Ethiopia	4	163	Y	3	150	17–78	76	50.7	
Ghana	1	49	Y	1	49	–	3	6.1	
Kenya	2	7	Y	0	0	–	–	–	
Libya	3	127	N	2	126	51–75	0	0	
Malawi	1	8	N	0	0	–	–	–	
Mali	1	47	N	1	47	–	0	0	
Mauritania	1	366	Y	1	366	–	6	1.6	
Mauritius	1	183	N	1	183	–	0	0	
Morocco	6	896	Y	4	890	51–398	47	5.3	
Mozambique	1	33	N	1	33	–	0	0	
Niger	1	21	Y	1	21	–	4	19	
Nigeria	32	1757	Y	21	1607	12–568	674	41.9	*mcr-1*
Rwanda	1	2473	Y	1	2473	–	35	1.4	
Sao Tome and Principe	1	1	Y	0	0	–	–	–	*mcr-1*
Senegal	1	33	Y	1	33	–	1	3	
Somalia	1	27	N	0	0	–	–	–	
South Africa	18	2665	Y	10	2605	16–683	98	3.8	*mcr-1*, mgrB, phoPQ/pmrAB
Sudan	1	71	Y	0	0	–	–	–	*mcr-1*
Tanzania	2	99	Y	1	30	–	0	0	*mcr-1*
Togo	3	80	Y	1	74	–	1	1.4	
Tunisia	15	15,852	Y	10	15,839	26–12,574	24	0.2	
Uganda	2	66	Y	1	61	–	10	16.4	
All reporting countries	158	29,301	Y	95	28,199	11–12,574	1096	3.9**	

*Y* one or more resistant isolates identified phenotypically or genotypically

*N* No resistant isolates identified phenotypically or genotypically

**Calculation should not be considered an estimate of overall resistance due to varying totals of specimens meeting criteria across nations

–Data not available

**Table 8:** Polymyxin (colistin and polymyxin B) resistance (R) and resistance determinants in *Klebsiella* spp. isolates: data from all years

				Findings in reports from all study years meeting criteria for generalizability					Identified resistance determinants
Nations	Number of reports	Specimens in all reports	Any R	Reports meeting criteria	Total specimens meeting criteria	Range of specimens among studies	Resistant specimens (#)	Resistant specimens (%)	
Algeria	17	2249	Y	11	2235	13–1184	1	< 0.1	*mcr-1*
Angola	1	23	N	0	0	–	–	–	
Benin	2	97	N	1	92	–	0	0	
Burkina Faso	3	262	Y	3	262	26–205	40	15.3	
Cameroon	3	41	Y	1	30	–	30	100	
Chad	1	18	N	1	18	–	0	0	
Congo	1	89	N	1	89	–	0	0	
Côte d'Ivoire	1	177	Y	1	177	–	14	7.9	
Djibouti	1	31	N	1	31	–	0	0	
Egypt	28	1276	Y	14	678	11–212	32	4.7	*mcr*-*1,* mgrB, phoPQ/pmrAB
Eritrea	1	14	N	0	0	–	–	–	
Ethiopia	4	163	Y	3	150	17–78	76	50.7	
Ghana	1	49	Y	1	49	–	3	6.1	
Kenya	2	7	Y	0	0	–	–	–	
Libya	3	127	N	2	126	51–75	0	0	
Malawi	1	8	N	0	0	–	–	–	
Mali	1	47	N	1	47	–	0	0	
Mauritania	1	366	Y	1	366	–	6	1.6	
Mauritius	1	183	N	1	183	–	0	0	
Morocco	6	896	Y	4	890	51–398	47	5.3	
Mozambique	1	33	N	1	33	–	0	0	
Niger	1	21	Y	1	21	–	4	19	
Nigeria	32	1757	Y	21	1607	12–568	674	41.9	*mcr-1*
Rwanda	1	2473	Y	1	2473	–	35	1.4	
Sao Tome and Principe	1	1	Y	0	0	–	–	–	*mcr-1*
Senegal	1	33	Y	1	33	–	1	3	
Somalia	1	27	N	0	0	–	–	–	
South Africa	18	2665	Y	10	2605	16–683	98	3.8	*mcr-1*, mgrB, phoPQ/pmrAB
Sudan	1	71	Y	0	0	–	–	–	*mcr-1*
Tanzania	2	99	Y	1	30	–	0	0	*mcr-1*
Togo	3	80	Y	1	74	–	1	1.4	
Tunisia	15	15,852	Y	10	15,839	26–12,574	24	0.2	
Uganda	2	66	Y	1	61	–	10	16.4	
All reporting countries	158	29,301	Y	95	28,199	11–12,574	1096	3.9**	

*Y* one or more resistant isolates identified phenotypically or genotypically

*N* No resistant isolates identified phenotypically or genotypically

**Calculation should not be considered an estimate of overall resistance due to varying totals of specimens meeting criteria across nations

–Data not available

#### Polymyxin resistance among more recent *E. coli* isolates

**Fig. 3:**
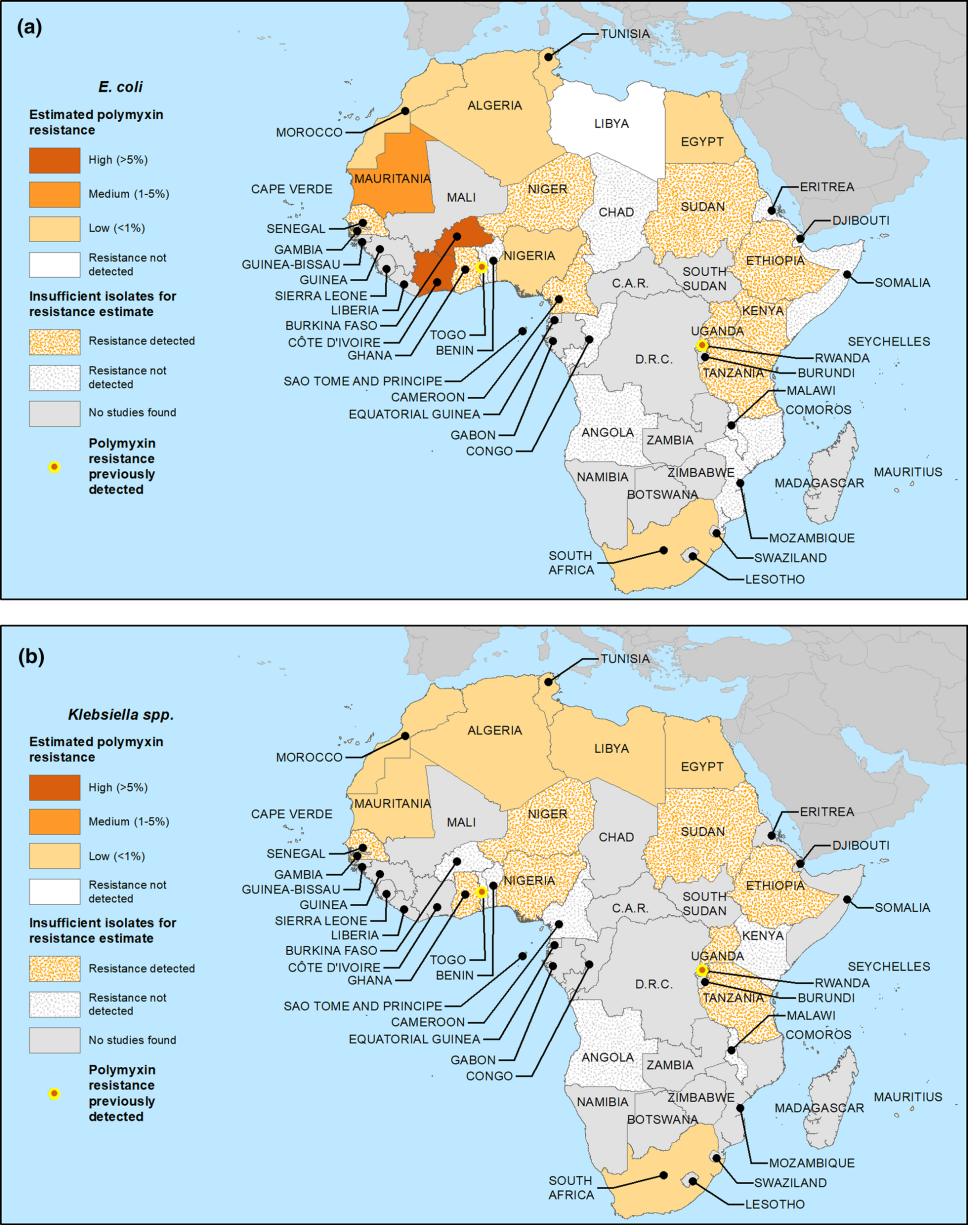
Estimated crude median national polymyxin(s) resistance proportions for **a** *E. coli* and **b** *Klebsiella* spp. for studies including samples from 2010 and later. For those nations with ≥ 100 isolates from qualifying studies (see Methods), median proportions across studies were calculated. Where < 100 isolates, data were deemed insufficient to estimate proportions and resistance is represented as either detected or not

Polymyxin resistance was identified among more recent *E. coli* isolates from 21 of 33 nations where either phenotypic or genotypic testing was performed (Table 9). Among 11 nations where at least 100 relevant *E. coli* isolates from 2010 onwards were tested, median polymyxin resistance was estimated as high in Burkina Faso and Côte d’Ivoire, moderate in Mauritania, low in Algeria, Egypt, Morocco, Nigeria, South Africa and Tunisia, and was not detected in Libya and Mauritius. Although resistance was detected, there were insufficient isolates to support estimates for 10 nations (Cameroon, Ethiopia, Ghana, Kenya, Niger, Sao Tome and Principe, Senegal, Sudan, Tanzania and Uganda). Similarly, there were 10 nations with insufficient *E. coli* isolates to support estimates where resistance was not detected (Angola, Benin, Chad, Congo, Djibouti, Eritrea, Malawi, Mozambique, Somalia and Togo). No relevant data were found from 18 nations (Botswana, Cape Verde, Central African Republic, Democratic Republic of the Congo, Equatorial Guinea, Gabon, Gambia, Guinea, Guinea-Bissau, Madagascar, Mali, Namibia, Rwanda, Sierra Leone, South Sudan, Zambia and Zimbabwe). Resistance data for *E. coli* are mapped in Fig. 3a.

**Table 9:** Polymyxin (colistin and polymyxin B) resistance (R) estimates and data for *Escherichia coli* isolates from studies including samples from 2010 and later

				Findings in reports from all study years meeting criteria for generalizability							Resistance estimate category
Nations	Number of reports	Specimens in all reports	Any R	Reports meeting criteria	Total specimens meeting criteria	Range of specimens among studies	Resistant specimens (#)	Resistant specimens (%)	Resistant range (%)	Median R	
Algeria	14	2168	Y	9	2155	13–1184	1	< 0.1	0–0.4	0	Low
Angola	1	23	N	0	0	-	-	-	-	-	Insufficient isolates—resistance not detected
Benin	2	97	N	1	92	-	0	0	-	N/A*	Insufficient isolates—resistance not detected
Burkina Faso	3	262	Y	3	262	26–205	40	15.3	0–61.3	10	High
Cameroon	3	41	Y	1	30	-	30	100	-	N/A*	Insufficient isolates—resistance detected
Chad	1	18	N	1	18	-	0	0	-	N/A*	Insufficient isolates—resistance not detected
Congo	1	89	N	1	89	-	0	0	-	N/A*	Insufficient isolates—resistance not detected
Côte d'Ivoire	1	177	Y	1	177	-	14	7.9	-	7.9	High
Djibouti	1	31	N	1	31	-	0	0	-	N/A*	Insufficient isolates—resistance not detected
Egypt	20	1015	Y	9	431	11–212	17	3.9	0–17.4	0.9	Low
Eritrea	1	14	N	0	0	-	-	-	-	-	Insufficient isolates—resistance not detected
Ethiopia	2	68	Y	1	55	-	50	90.9	-	N/A*	Insufficient isolates—resistance detected
Ghana	1	49	Y	1	49	-	3	6.1	-	N/A*	Insufficient isolates—resistance detected
Kenya	2	7	Y	0	0	-	-	-	-	-	Insufficient isolates—resistance detected
Libya	3	127	N	2	126	51–75	0	0	0–0	0	Resistance not detected
Malawi	1	8	N	0	0	-	-	-	-	-	Insufficient isolates—resistance not detected
Mauritania	1	366	Y	1	366	-	6	1.6	-	1.7	Moderate
Mauritius	1	183	N	1	183	-	0	0	-	0	Resistance not detected
Morocco	5	893	Y	4	890	51–398	47	5.3	0–11.3	0.3	Low
Mozambique	1	33	N	1	33	-	0	0	-	N/A*	Insufficient isolates—resistance not detected
Niger	1	21	Y	1	21	-	4	19	-	N/A*	Insufficient isolates—Resistance detected
Nigeria	8	125	Y	3	111	18–50	1	0.9	0–2.3	0	Low
Sao Tome and Principe	1	1	Y	0	0	-	-	-	-	-	Insufficient isolates—resistance detected
Senegal	1	33	Y	1	33	-	1	3	-	N/A*	Insufficient isolates—Resistance detected
Somalia	1	27	N	0	0	-	-	-	-	-	Insufficient isolates—resistance not detected
South Africa	14	2005	Y	6	1945	16–683	12	0.6	0–0.9	0.15	Low
Sudan	1	71	Y	0	0	-	-	-	-	-	Insufficient isolates—resistance detected ^
Tanzania	2	99	Y	1	30	-	0	0	-	N/A*	Insufficient isolates—resistance detected
Togo	2	6	N	0	0	-	-	-	-	-	Insufficient isolates—Resistance not detected
Tunisia	11	3014	Y	7	3002	26–1075	13	0.4	0–1.3	0	Low
Uganda	2	66	Y	1	61	-	10	16.4	-	N/A*	Insufficient isolates—resistance detected
All reporting countries	109	11,137	Y	58	10,190	11–1184	249	2.4**	–	–	–

*Y* one or more resistant isolates identified phenotypically or genotypically

*N* no resistant isolates identified phenotypically or genotypically

^Only genotypic resistance reported

*Insufficient isolates (< 100) for polymyxin resistance estimate

**Calculation should not be considered an estimate of overall resistance due to varying totals of specimens meeting criteria across nations

-Data not available

–Not calculated

#### Polymyxin resistance among more recent *Klebsiella* isolates

Polymyxin resistance was identified among more recent *Klebsiella* isolates from 18 of 24 nations where either phenotypic or genotypic testing was performed (Table 10). Resistance was detected in all 8 nations with at least 100 generalizable *Klebsiella* isolates studied (Algeria, Egypt, Libya, Mauritania, Mauritius, Morocco, South Africa and Tunisia), and was estimated as low in each. Among nations with insufficient isolates to support a resistance estimate, resistance was detected in 8 (Ethiopia, Ghana, Niger, Nigeria, Senegal, Sudan, Tanzania and Uganda) and not detected in 7 (Angola, Benin, Burkina Faso, Cameroon, Kenya, Malawi and Togo). No studies were identified from 25 nations (Botswana, Cape Verde, Central African Republic, Chad, Congo, Côte d’Ivoire, Democratic Republic of the Congo, Djibouti, Equatorial Guinea, Eritrea, Gabon, Gambia, Guinea, Guinea-Bissau, Madagascar, Mali, Mozambique, Namibia, Rwanda, Sao Tome and Principe, Sierra Leone, Somalia, South Sudan, Zambia and Zimbabwe). Resistance data for *Klebsiella* are mapped in Fig. 3b.

**Table 10:** Polymyxin (colistin and polymyxin B) resistance (R) estimates and data for *Klebsiella* spp. isolates from studies including samples from 2010 and later

				Findings in reports from all study years meeting criteria for generalizability							Resistance estimate category
Nations	Number of reports	Specimens in all reports	Any R	Reports meeting criteria	Total specimens meeting criteria	Range of specimens among studies	Resistant specimens (#)	Resistant specimens (%)	Resistant range (%)	Median R	
Algeria	17	1056	Y	6	1015	13–608	2	0.2	0–4.3	0	Low
Angola	1	24	N	0	0	-	-	-	-	-	Insufficient isolates—resistance not detected
Benin	2	51	N	2	51	10–41	0	0	0–0	N/A*	Insufficient isolates—resistance not detected
Burkina Faso	1	5	N	0	0	-	-	-	-	-	Insufficient isolates—resistance not detected
Cameroon	1	3	N	0	0	-	-	-	-	-	Insufficient isolates—resistance not detected
Egypt	27	1084	Y	10	605	14–183	11	1.8	0–50	0	Low
Ethiopia	4	53	Y	3	51	10–30	17	33.3	0–90	N/A*	Insufficient isolates—resistance detected
Ghana	1	38	Y	1	38	-	5	13.2	-	N/A*	Insufficient isolates—Resistance detected
Kenya	1	5	N	0	0	-	-	-	-	-	Insufficient isolates—Resistance not detected
Libya	8	179	Y	3	136	24–76	6	4.4	0–25	0	Low
Malawi	2	8	N	0	0	-	-	-	-	-	Insufficient isolates—resistance not detected
Mauritania	1	137	Y	1	137	-	1	0.7	-	0.8	Low
Mauritius	2	119	Y	1	118	-	0	0	-	0	Low
Morocco	7	245	Y	4	232	10–118	28	12.1	0–22.9	0.6	Low
Niger	1	4	Y	0	0	-	-	-	-	-	Insufficient isolates—resistance detected
Nigeria	9	95	Y	4	80	10–32	0	0	0–0	N/A *	Insufficient isolates—Resistance detected
Senegal	1	34	Y	1	34	-	3	8.8	-	N/A*	Insufficient isolates—resistance detected
South Africa	15	2286	Y	7	1682	10–839	18	1.1	0–2.9	0	Low
Sudan	1	50	Y	0	0	-	-	-	-	-	Insufficient isolates—resistance detected^
Tanzania	2	59	Y	0	0	-	-	-	-	-	Insufficient isolates—resistance detected
Togo	2	31	N	1	30	-	0	0	-	N/A*	Insufficient isolates—resistance not detected
Tunisia	20	4250	Y	11	4148	11–2826	60	1.4	0–6.2	0.5	Low
Uganda	2	20	Y	1	12	-	3	25	-	N/A*	Insufficient isolates—resistance detected
All reporting countries	128	9836	Y	56	8369	10–2826	154	1.8**	–	–	–

*Y* one or more resistant isolates identified phenotypically or genotypically

*N* no resistant isolates identified phenotypically or genotypically

^Only genotypic resistance reported

*Insufficient isolates (< 100) for polymyxin resistance estimate

**Calculation should not be considered an estimate of overall resistance due to varying totals of specimens meeting criteria across nations

-Data not available

–Not calculated

#### Polymyxin resistance genotypes

Genotypic determinants of polymyxin resistance in *E. coli* were characterized in 15 data reports on isolates from 7 nations (Table 7 and Fig. 2), with *mcr-1* found in all (Algeria, Egypt, Nigeria, Sao Tome and Principe, South Africa, Sudan and Tanzania). *phoPQ*/*pmrAB* and *mgrB* were identified in *E. coli* from Egypt and South Africa. Among *Klebsiella*, genotypic polymyxin resistance determinants were identified in 12 reports on isolates from 7 nations (Table 8 and Fig. 2). *mcr-1* was identified in Egypt, South Africa and Sudan, and *mcr-8* in Algeria. *mgrb* was reported from six nations (Algeria, Egypt, Libya, Nigeria, South Africa and Tunisia), and *phoPQ*/*pmrAB* identified from 4 (Algeria, Egypt, South Africa and Tunisia).

#### Documented geographic overlaps of carbapenem and polymyxin resistance

Overlapping resistance to carbapenems and polymyxin(s) among *E. coli* or *Klebsiella*, whether phenotypic and/or genotypic, was documented in 23 nations with overlapping genotypic resistance present in 9 (Fig. 2). Specific geographic overlaps between NDM carbapenemases and *mcr* genetic determinants were identified in 6 nations (Algeria, Egypt, Nigeria, South Africa, Sudan and Tanzania).

## Discussion

We searched for and conducted meta-analyses and mapping of available data on carbapenem and polymyxin resistance in *E. coli* and *Klebsiella* isolates from humans in Africa. These analyses, which included 1479 unique data reports through the end of 2019, show that resistance to each of these important antibiotic classes has become increasingly widespread on the continent.

The availability of a large amount of additional data since our prior report on WHO Africa nations [2] provided substantive new insights into the distribution of carbapenem resistance and its genotypic determinants, with resistance documented in approximately ¾ of African nations (compared to less than half previously for WHO Africa [2]). Carbapenem resistance among *Klebsiella* was significant in most countries with sufficient isolates to support a resistance estimate and categorized as high in 10, and moderate and low in 6 nations respectively. Among *E. coli*, estimated resistance was generally somewhat lower: high in 3, moderate in 7, and low in 14 nations with sufficient isolates. Levels of carbapenem resistance appeared high in contiguous areas of Northern and Eastern Africa (e.g. for *Klebsiella* in Libya, Egypt, Sudan, Ethiopia and Kenya, Fig. 1b). The most widespread genes conferring carbapenem resistance in both species, including in that area, were *bla*_OXA-48_, *bla*_NDM-1_ and *bla*_OXA-181_. Taken together, the analyses document continuing continent-wide spread of carbapenem resistance and of a broad variety of transferrable resistance plasmids, raising concerns about the future reliability of carbapenems.

Given their importance in treating resistant infections, and the paucity of available data, we also searched for and analyzed available information on polymyxin susceptibility. We located data on polymyxin susceptibility for *E. coli* and/or *Klebsiella* spp. isolates from 33 of 54 African nations, with resistance identified in 23 of those 33 nations (69.7%) from which any data were available. For the small minority of nations with ≥ 100 isolates studied from 2010 and later, estimated resistance among *E. coli* to polymyxins was high in 2, moderate in 1 and low in 6. Although resistance was estimated as high in two nations, estimates were based on relatively limited isolate and study numbers, and, in many cases, older methods of susceptibility testing, and should be interpreted with caution. Estimated resistance to polymyxins was low among *Klebsiella* in all 8 nations with sufficient isolates to support an estimate. Polymyxin resistance genetic determinants were evaluated among *E. coli* and *Klebsiella* in 7 nations each, with the mobile *mcr-1* determinant shown to be predominant, consistent with recent reviews of the genetics of colistin resistance in *E. coli* both globally [35] and in Africa [36].

Our analyses also show, even based on limited information available from many areas (particularly with respect to polymyxins), that geographic overlapping of carbapenem and polymyxin resistance has become common and widespread, with 23 nations having documented phenotypic and/or genotypic resistance for both. Furthermore, overlapping plasmid mediated resistance to the two drug classes was documented in 9 nations, including the presence of both NDM carbapenemases and *mcr* genetic determinants in 6. These findings document highly concerning ongoing risks from transferrable resistance, including, were *bla*_NDM_ and *mcr* to be acquired by the same organism(s), the risk of infections not susceptible to currently available antibiotics.

Despite efforts to enhance surveillance, major information gaps remain. For example, searches yielded no data on polymyxin resistance from 21 nations, and 6 nations with no available data on carbapenem resistance. Furthermore, even from countries where data were available, there were often less than 100 recent isolates studied, not meeting minimal pre-specified criteria to support crude estimation of resistance proportions.

It is important to note a number of limitations of these analyses, discussed in detail previously [2, 31]. Despite use of predefined study inclusion criteria and employment of common data elements, the inherently diverse data sources, time periods and locations, as well as study designs and methods, mean that inferences must be made with caution and the data should be interpreted in the context of the timing, location and populations studied. Interested readers can access further details, including the primary data from individual reports on specific nations, in the supplemental material (Additional file 4). In addition, susceptibility testing methods and standards for breakpoints to interpret their results have evolved considerably over time and often differ among laboratories. Therefore, comparability of results across laboratories, nations and time periods may be affected by such differences. For carbapenems, minimum inhibitory concentrations considered susceptible have decreased over time, meaning that some decrease in the proportion of isolates susceptible may be expected due to changing standards. There are also major caveats with respect to the interpretation of reported polymyxin susceptibility testing results. Rather than utilizing currently recommended broth microdilution methods, most studies were performed using previously employed disk diffusion methods which may be inconsistent and may overestimate susceptibility. Therefore, while the presence and spread of resistance to polymyxins is well documented, often at both phenotypic and genotypic levels, rate estimates must be interpreted with caution.

Looking at the totality of the data, despite well over a thousand data reports from hundreds of studies, the available information from many countries was limited or, in some cases, absent. Additionally, lag periods between data acquisition and reporting, along with the analysis time since the searches included in the current study, which utilized data available through December 31, 2019, mean that the continued documentation and spread of resistance to new areas is fully expected. Thus, the non-detection of resistance in a nation should not be considered as evidence that resistance was or is absent. Ensuring a more complete picture of resistance distribution and rates will require both ongoing surveillance and continued updating of data and analyses. As also noted, where resistance proportions have been estimated, these should generally be considered to be crude approximations based on non-random reporting and samples, although in our prior study of Southeast Asia [31] the results from similarly performed meta-analyses generally tracked with national surveillance where available. Similarly, available genotypic data are even more limited, with laboratories often assaying for a limited number of specific genotype(s) rather than broadly characterizing isolates with multiplex or sequence-based methods, likely leading to under-detection of less recognized or uncommon genotypes. Other potential factors may also affect the representativeness of the data, including the tendency toward publication of positive results and the likelihood that laboratories performing susceptibility testing may be located in more urban and regional centers, typically associated with more complex care and drug resistance. We attempted to address such issues by searching not only for positive but also for negative results such as in publications where susceptibility testing was reported but not as the focus of the studies.

Despite such limitations, the findings show the widespread and overlapping presence of carbapenem and polymyxin resistance among *E. coli* and *Klebsiella* isolates from humans in Africa and highlight the urgent need to better address remaining gaps in surveillance, including to systematically determine and track rates of carbapenem and polymyxin resistance, and to monitor for the emergence of dually resistant organisms. To do so will require adequate support for sustainable laboratory and epidemiologic capacity, as stressed by both WHO [41] and the African Union and Africa CDC [42]. Robust ongoing longitudinal AMR surveillance is also critical to inform antibiotic stewardship initiatives [41, 43]. Furthermore, the widespread nature of the CRE and polymyxin resistance threats reinforces the importance of strong infection prevention and control in healthcare facilities [41, 44]. Beyond enhanced stewardship of antimicrobials and measures to contain the spread of MDRO in healthcare, the continuing use of important antimicrobials, including colistin, in animal production remains a problem that must be fully addressed [45]. Resistant organisms may also be present in and spread through waste water, including from healthcare facilities [46], agriculture, and aquaculture [46].

## Conclusions

Carbapenem resistance among *E. coli* and *Klebsiella* is widely distributed in Africa, and documented in 40 of 54 nations. Although resistance rates for nations with sufficient isolates to support estimates were typically low to moderate, high rates (> 5%) were found in several nations, including 10 nations with high rates among *Klebsiella*. Although far less data are available concerning polymyxins, resistance was documented in 23 of 33 nations with available data. The most widespread resistance associated genotypes were, for carbapenems, *bla*_OXA-48_, *bla*_NDM-1_ and *bla*_OXA-181_ and, for polymyxins, *mcr-1*, *mgrB*, and *phoPQ*/*pmrAB*. Overlapping phenotypic and/or genotypic resistance to both carbapenems and polymyxins was documented in 23 nations, including the presence of both transferrable NDM carbapenemases and *mcr* determinants of polymyxin resistance in 6. These findings point to ongoing and significant risks to patient safety and public health from carbapenem and polymyxin resistance. Despite progress in recent years, resistance appears to be spreading and numerous data gaps remain, indicating the need to fully support robust AMR surveillance, antimicrobial stewardship and infection control in a manner that also addresses animal and environmental health dimensions. A One Health approach that enhances surveillance and reduces both the inappropriate use of critical antibiotics and the spread of resistant organisms in all relevant settings is essential [47].

### Supplementary Information

**Additional file 1**: Boolean search strings constructed for searches of scientific databases.

**Additional file 2**: Study flow diagram.

**Additional file 3**: Annotation on data entry columns and abbreviations.

**Additional file 4**: Study data.

## Data Availability

The dataset supporting the conclusions of this article is available in the Harvard Dataverse repository, https://doi.org/10.7910/DVN/JIJH3W. The dataset(s) supporting the conclusions of this article is also included within the article as Additional file 4.
