# Correction: Review and analysis of the overlapping threats of carbapenem and polymyxin resistant E. Coli and Klebsiella in Africa

**DOI:** 10.1186/s13756-024-01403-7

**Published:** 2024-05-12

**Authors:** Danielle M. Venne, David M. Hartley, Marissa D. Malchione, Michala Koch, Anjali Y. Britto, Jesse L. Goodman

**Affiliations:** 1 Center on Medical Product Access, Safety and Stewardship, Georgetown University, 3900 Reservoir Road, Washington, DC 20057 USA; 2 James M. Anderson Center for Health Systems Excellence, Cincinnati Children’s Hospital, 3333 Burnet Avenue, Cincinnati, OH 45229 USA; 3 Department of Pediatrics, College of Medicine, University of Cincinnati, Cincinnati, OH 45229 USA; 4 Sabin Vaccine Institute, Influenza Vaccine Innovation, 2175 K St NW, Washington, DC 20037 USA

**Correction to Antimicrobial Resistance & Infection Control (2023) 12:29**

10.1186/s13756-023-01220-4

The original article contains two errors that the authors wish to note corrections for:

1. In Table 2, the four cells in the ‘All reporting nations’ each display incorrect values, and should each instead be displayed as in the amended Table 1 ahead.
2. In Additional File 1, a search term was inadvertently repeated when the intent was to include a special character, and there is an additional inadvertent repetition of a term. The corrected Additional File 1 can be viewed via this Correction article.

**Table 2:** Available reports on *E. coli* and *Klebsiella* carbapenem and polymyxin susceptibility, resistance, and related genes

Nation	All reports on named species (reports identifying resistance or determinants related to resistance)				
	Carbapenem		Polymyxin (colistin and polymyxin B)		
	*E. coli*	*Klebsiella*	*E. coli*	*Klebsiella*	References
Algeria	33 (12)	37 (18)	17 (4)	22 (3)	[48–97]
Angola	2 (2)	2 (2)	1 (0)	1 (0)	[98,99]
Benin	6 (4)	2 (1)	2 (0)	2 (0)	[100–105]
Botswana	1 (0)	1 (0)	0	0	[106]
Burkina Faso	12 (4)	13 (0)	3 (2)	1 (0)	[25,107–121]
Cameroon	11 (3)	7 (3)	3 (2)	1 (0)	[116,122–133]
Cape Verde	1 (0)	0	0	0	[116]
Central African Republic	2 (0)	3 (0)	0	0	[25,134,135]
Chad	8 (4)	4 (1)	1 (0)	0	[57,116,136–141]
Congo	2 (2)	1 (1)	1 (0)	0	[142,143]
Côte d’Ivoire	4 (1)	5 (1)	1 (1)	0	[144–150]
Democratic Republic of the Congo	3 (0)	2 (1)	0	0	[151–153]
Djibouti	2 (1)	0	1 (0)	0	[57,154]
Egypt	106 (66)	125 (98)	28 (14)	34 (15)	[1,25–27,57,78,116,155–293]
Equatorial Guinea	1 (0)	1 (1)	0	0	[294]
Eritrea	1 (0)	0	1 (0)	0	[295]
Ethiopia	19 (10)	27 (17)	4 (4)	6 (4)	[1,112,296–316]
Gabon	4 (0)	5 (1)	0	0	[317–321]
Gambia	1 (1)	1 (1)	0	0	[322]
Ghana	15 (5)	15 (8)	1 (1)	1 (1)	[116,323–337]
Guinea	1 (0)	0	0	0	[116]
Guinea-Bissau	1 (0)	1 (0)	0	0	[338]
Kenya	26 (11)	25 (21)	2 (1)	2 (0)	[116,168,268,282,339–362]
Libya	17 (10)	22 (20)	3 (0)	8 (4)	[57,78,363–385]
Madagascar	14 (3)	12 (6)	0	0	[1,26,27,112,116,168,386–395]
Malawi	4 (3)	7 (5)	1 (0)	2 (0)	[26,27,396–398]
Mali	4 (3)	2 (1)	1 (0)	0	[1,57,399,400]
Mauritania	1 (1)	1 (0)	1 (1)	1 (1)	[401]
Mauritius	4 (2)	6 (5)	1 (0)	2 (1)	[25,57,168,183,402–404]
Morocco	24 (13)	39 (24)	6 (2)	10 (2)	[25,57,78,116,183,268,282,405–433]
Mozambique	6 (1)	4 (0)	1 (0)	0	[1,116,427,434–438]
Namibia	1 (0)	5 (1)	0	0	[25,183,439]
Niger	5 (2)	2 (0)	1 (1)	1 (1)	[57,440–443]
Nigeria	82 (53)	80 (46)	32 (23)	28 (15)	[1,27,116,444–561]
Rwanda	7 (3)	6 (2)	1 (1)	1 (1)	[562–568]
Sao Tome and Principe	1 (1)	1 (1)	1 (1)	0	[569]
Senegal	10 (2)	11 (6)	1 (1)	3 (1)	[56,78,112,116,145,570–581]
Sierra Leone	5 (3)	4 (3)	0	0	[116,582,583]
Somalia	1 (0)	0	1 (0)	0	[295]
South Africa	69 (25)	109 (82)	18 (8)	20 (10)	[1,25,78,168,183,268,282,519,584–663]
South Sudan	1 (0)	0	0	0	[664]
Sudan	12 (7)	10 (5)	1 (1)	1 (1)	[1,27,112,116,665–673]
Tanzania	29 (6)	26 (7)	2 (1)	2 (1)	[56,112,116,168,674–700]
Togo	6 (4)	4 (3)	3 (1)	3 (1)	[116,701–706]
Tunisia	34 (14)	70 (43)	15 (4)	29 (13)	[1,26,27,78,168,183,268,282,707–774]
Uganda	17 (10)	16 (11)	2 (1)	2 (1)	[1,26,27,168,775–788]
Zambia	3 (1)	4 (4)	0	0	[26,27,789,790]
Zimbabwe	3 (1)	1 (1)	0	0	[116,791,792]
All reporting nations	622 (294)	719 (451)	158 (75)	183 (76)	

Reports on carbapenem or polymyxin susceptibility were not identified from the following searched nations: Burundi, Comoros, Lesotho, Liberia, Seychelles and Swaziland

### Electronic supplementary material

Below is the link to the electronic supplementary material.

Supplementary Material 1

