# Supplementary material for: Review and analysis of the overlapping threats of carbapenem and polymyxin resistant E. coli and Klebsiella in Africa
Source: Antimicrob Resist Infect Control. 2023 Apr 4;12:29. doi: 10.1186/s13756-023-01220-4 (PMC10071777; doi:10.1186/s13756-023-01220-4)
Supplement: Supplementary file 2 — Additional file 2: Study flow diagram. [file 13756_2023_1220_MOESM2_ESM.docx]

**Additional file 2. Study flow diagram.**

Additional records identified, reviewed and selected through other sources

[records identified through citation search (meta-analyses, systematic reviews and selected studies), Google Scholar searches and original manuscript]

*n* = 907

Records identified through database searching

Embase = 783

Global Health = 1832

PubMed = 2268

Web of Science = 2841

Full-text articles assessed and excluded

*n* = 439

-insufficient study detail

-incorrect geography

-incorrect organism

-insufficient detail on testing

Records screened after

non-relevant material/duplications removed

*n* = 1191

Studies included in qualitative synthesis

*n* = 749

Unique data reports deemed ‘generalizable’

(see Methods)

Carbapenem studies = 1341

Colistin/Polymyxin B studies = 341

Unique data reports included in qualitative synthesis

*n* = 1479
