# Supplementary material for: Review and analysis of the overlapping threats of carbapenem and polymyxin resistant E. coli and Klebsiella in Africa
Source: Antimicrob Resist Infect Control. 2023 Apr 4;12:29. doi: 10.1186/s13756-023-01220-4 (PMC10071777; doi:10.1186/s13756-023-01220-4)
Supplement: Supplementary file 3 — Additional file 3: Annotation on data entry columns and abbreviations. [file 13756_2023_1220_MOESM3_ESM.docx]

**Additional file 3. Annotation on data entry columns and abbreviations.**

REF: Reference number as cited in text and reference list

LAT, LONG: Latitude and longitude

FACILITY: Facility where isolates collected

CITY: City where isolates collected

PROVINCE_OR_STATE: Province or State where isolates collected

REGION: WHO region

PUB: Year of publication

START, END: Dates sampling initiated and ended

DUR: Duration of sample collection in months

SITE: Study was performed at single site (SINGLE), multiple sites (MULTI), or an unknown number of sites (UNK)

POP_1, _2, _3: Populations included in the study sample: healthcare acute (HC-ACUTE), healthcare chronic (HC-LONG), healthcare unknown (HC-UNK), community (COMM), and TRAVELERS (see study Methods for details)

SUBPOP: Selected subpopulations, if studied (e.g., pregnant, ICU, clinical syndrome etc.)

AGE_1, _2, _3: Age groups included (e.g. ADOLESCENT, ADULT, ELDERLY etc.), as reported by author(s) or, where possible to ascertain, per WHO classification (see study Methods for details)

SAMPLE_ 1, _2: Types of samples from which cultures derived (e.g. BLOOD, URINE, STOOL, etc.), multiple (MULT), and unknown (UNK)

DATA TYPE_1, _2: (CLIN LAB) = derived from series of clinical laboratory samples used for diagnosis, (CASE SERIES) = focused on a specific subpopulation of patients where clinical information was included beyond demographics, (OUTBREAK) = specimens obtained during an outbreak of resistant infections, (SURVEILLANCE) = studies evaluating susceptibility among colonizing organisms, rather than samples obtained for clinical diagnosis (see study Methods for details)

SELECT ISOLATES: specific subsets of laboratory isolates selected for their resistance (e.g. ESBL, Carbapenem – R, MDR etc.)

SPECIES: *Escherichia coli* (*E. coli*), *Klebsiella michiganensis* (*K. mich*), *Klebsiella ornithinolytica* (*K. orni*), *Klebsiella oxytoca* (*K. oxy*), *Klebsiella ozaenae* (*K.* *ozae*), *Klebsiella ozonea* (*K.* *ozo*), *Klebsiella planticola* (*K. plan*), *Klebsiella pneumoniae* *(K. pneu*), *Klebsiella variicola* (*K. vari*), and *Klebsiella* species (*K.* spp)

CARB_RESIST: (R) = carbapenem resistance detected in at least one sample studied, (S) = no carbapenem resistance detected in any samples

TOTAL: Total number of isolates studied

ERTA_R, DORI_R, IMI_R, MERO_R, CARB_NOS: Per cent of samples studied resistant to ertapenem, doripenem, imipenem, meropenem or carbapenem not otherwise specified.

COL_POLY_M_B_RESIST: (R) = colistin or polymyxin B resistance detected in at least one sample studied, (S) = no colistin or polymyxin B resistance detected in any samples

COL_POLYM_B_R: Per cent of samples studied resistant to colistin or polymyxin B

CARB_DENOM_Y_NO: (Y) = data report for carbapenem susceptibility testing met criteria for generalizability and included in quantitative analyses. (N) = data report for carbapenem susceptibility testing did not meet criteria for generalizability and is not included in quantitative analyses

COL_POLYM_B_DENOM_Y_NO: (Y) = data report for colistin and/or polymyxin B susceptibility testing met criteria for generalizability and included in quantitative analyses. (N) = data report for colistin and/or polymyxin B susceptibility testing did not meet criteria for generalizability and is not included in quantitative analyses

GES, IMI, IMP_unspecified, IMP_1, KPC_unspecified, KPC_2, KPC_3, NDM_unspecified, NDM_1, NDM_4, NDM_5, NDM_6, NDM_7, OMP, OXA_unspecified, OXA_23, OXA_48, OXA_58, OXA_181, OXA_232, VIM_unspecified, VIM_1, VIM_2, VIM_4, VIM_19, MCR_unspecified, MCR_1, MCR_2, MCR_3,MCR_4, MCR_5, MCR_8, mgrB_mutation, PhoPQ_PmrAB_mut: (Y) = genotype/mutation detected, (N) = genotype/mutation tested for but not detected

DATABASE = Database(s) or source(s) of grey literature (e.g. non-peer reviewed and/or Internet based) where study identified
