# Supplementary material for: Correction: Review and analysis of the overlapping threats of carbapenem and polymyxin resistant E. Coli and Klebsiella in Africa
Source: Antimicrob Resist Infect Control. 2024 May 12;13:51. doi: 10.1186/s13756-024-01403-7 (PMC11089759; doi:10.1186/s13756-024-01403-7)
Supplement: Supplementary file 1 — Supplementary Material 1 [file 13756_2024_1403_MOESM1_ESM.docx]

**Additional file 1. Boolean search strings constructed for searches of scientific databases.**

| **Search strings**  General structure: (Africa AND (Resistance OR Drug) AND (Infection OR Enzyme OR genotype) | |
| --- | --- |
| **PubMed**  **Filters: From 2017/01/01 to 2019/12/31 and Humans** | |
| **Place** | Search ((Africa OR Algeria OR Angola OR Benin OR Botswana OR "Burkina Faso" OR Burundi OR Cameroon OR "Cabo Verde" OR ("Central African Republic" OR CAR) OR Chad OR Comoros OR Congo OR ("Cote d'Ivoire" OR "Ivory Coast") OR "Democratic Republic of the Congo" OR "Equatorial Guinea" OR Eritrea OR Ethiopia OR Gabon OR Gambia OR Ghana OR Guinea OR "Guinea-Bissau" OR Kenya OR Lesotho OR Liberia OR Madagascar OR Malawi OR Mali OR Mauritania OR Mauritius OR Mozambique OR Namibia OR Niger OR Nigeria OR Rwanda OR "Sao Tome and Principe" OR Senegal OR Seychelles OR "Sierra Leone" OR "South Africa" OR "South Sudan" OR Swaziland OR Togo OR Uganda OR ("United Republic of Tanzania" OR Tanzania) OR Zambia OR Zimbabwe)) |
|  | **AND** |
| **Resistance OR Drug** | (("antimicrobial resistance" OR "antimicrobial resistant" OR AMR) OR ("Drug Resistance"[Mesh]) OR (Drug Resistance, Bacterial[Mesh]) OR ("multidrug resistance" OR "multidrug resistant" OR MDR) OR ("multiple antibiotic resistance" OR MAR[Title/Abstract]) OR (antibiotic AND resistan*) OR (resistan*) OR (carbapenem* OR carbapenems OR imipenem OR imipenems OR doripenem OR doripenems OR meropenem OR meropenems OR ertapenem OR ertapenems)) |
|  | **AND** |
| **Infection OR Enzyme OR genotype** | (("Gram-Negative Bacterial Infections"[Mesh]) OR "Healthcare-associated infection" OR "Cross-Infection"[Mesh] OR "community-associated infection" OR "Community-Acquired Infections"[Mesh] OR nosocomial OR (ESBL OR "ESBL isolates" OR "extended-spectrum β-lactamases" OR "extended-spectrum beta-lactamases") OR ("Enterobacteriaceae"[Mesh] OR "Enterobacteriaceae Infections"[Mesh]) OR ("carbapenem-resistant enterobacteriaceae" OR CRE OR "Carbapenem resistant enterobacteria*" OR carbapenem resistan* OR carbapenem) OR "Enterobacter" OR "Klebsiella" OR "Escherichia" OR (carbapenemase OR carbapenemases OR KPC OR GES OR IMP OR NDM OR OXA OR VIM)))) |

|  | |
| --- | --- |
| **PubMed**  **Filters: From 1996/01/01 to 2019/12/31 and Humans** | |
| **Place** | Search ((Africa OR Djibouti OR Egypt OR Libya OR Morocco OR Somalia OR Sudan OR Tunisia)) |
|  | **AND** |
| **Resistance OR Drug** | (("antimicrobial resistance" OR "antimicrobial resistant" OR AMR) OR ("Drug Resistance"[Mesh]) OR (Drug Resistance, Bacterial[Mesh]) OR ("multidrug resistance" OR "multidrug resistant" OR MDR) OR ("multiple antibiotic resistance" OR MAR[Title/Abstract]) OR (antibiotic AND resistan*) OR (resistan*) OR (carbapenem* OR carbapenems OR imipenem OR imipenems OR doripenem OR doripenems OR meropenem OR meropenems OR ertapenem OR ertapenems)) |
|  | **AND** |
| **Infection OR Enzyme OR genotype** | (("Gram-Negative Bacterial Infections"[Mesh]) OR "Healthcare-associated infection" OR "Cross-Infection"[Mesh] OR "community-associated infection" OR "Community-Acquired Infections"[Mesh] OR nosocomial OR (ESBL OR "ESBL isolates" OR "extended-spectrum β-lactamases" OR "extended-spectrum beta-lactamases") OR ("Enterobacteriaceae"[Mesh] OR "Enterobacteriaceae Infections"[Mesh]) OR ("carbapenem-resistant enterobacteriaceae" OR CRE OR "Carbapenem resistant enterobacteria*" OR carbapenem resistan* OR carbapenem) OR "Enterobacter" OR "Klebsiella" OR "Escherichia" OR (carbapenemase OR carbapenemases OR KPC OR GES OR IMP OR NDM OR OXA OR VIM)))) |

|  | |
| --- | --- |
| **PubMed**  **Filters: From 1996/01/01 to 2019/12/31 and Humans** | |
| **Place** | Search: ((Africa OR Algeria OR Angola OR Benin OR Botswana OR "Burkina Faso" OR Burundi OR Cameroon OR "Cabo Verde" OR ("Central African Republic" OR CAR) OR Chad OR Comoros OR Congo OR ("Cote d'Ivoire" OR "Ivory Coast") OR "Democratic Republic of the Congo" OR Djibouti OR Egypt OR "Equatorial Guinea" OR Eritrea OR Ethiopia OR Gabon OR Gambia OR Ghana OR Guinea OR "Guinea-Bissau" OR Kenya OR Lesotho OR Liberia OR Libya OR Madagascar OR Malawi OR Mali OR Mauritania OR Mauritius OR Morocco OR Mozambique OR Namibia OR Niger OR Nigeria OR Rwanda OR "Sao Tome and Principe" OR Senegal OR Seychelles OR "Sierra Leone" OR Somalia OR "South Africa" OR "South Sudan" OR Sudan OR Swaziland OR Togo OR Uganda OR ("United Republic of Tanzania" OR Tanzania) OR Tunisia OR Zambia OR Zimbabwe)) |
|  | **AND** |
| **Resistance OR Drug** | (("antimicrobial resistance" OR "antimicrobial resistant" OR AMR) OR ("Drug Resistance"[Mesh]) OR (Drug Resistance, Bacterial[Mesh]) OR ("multidrug resistance" OR "multidrug resistant" OR MDR) OR ("multiple antibiotic resistance" OR MAR[Title/Abstract]) OR (antibiotic AND resistan*) OR (resistan*) OR Colistin OR polymyxin OR "polymyxin E" OR "polymyxin B")) |
|  | **AND** |
| **Infection OR Enzyme OR genotype** | ((Gram-Negative Bacterial Infections"[Mesh]) OR "Healthcare-associated infection" OR "Cross-Infection"[Mesh] OR "community-associated infection" OR "Community-Acquired Infections"[Mesh] OR nosocomial OR (ESBL OR "ESBL isolates" OR "extended-spectrum β-lactamases" OR "extended-spectrum beta-lactamases") OR ("Enterobacteriaceae"[Mesh] OR "Enterobacteriaceae Infections"[Mesh]) OR ("polymyxin resistant Enterobacteriaceae") OR ("colistin resistant Enterobacteriaceae") OR "Enterobacter" OR "Klebsiella" OR "Escherichia" OR (mcr OR "mcr-1" OR "mcr-2" OR "mcr-3”)) |

| **Web of Science**  **Filters: From 2017-2019 and English** | |
| --- | --- |
| **Place** | (TS=("Africa" OR Algeria OR Angola OR Benin OR Botswana OR "Burkina Faso" OR Burundi OR Cameroon OR "Cabo Verde" OR ("Central African Republic" OR CAR) OR Chad OR Comoros OR Congo OR ("Cote d'Ivoire" OR "Ivory Coast") OR "Democratic Republic of the Congo" OR "Equatorial Guinea" OR Eritrea OR Ethiopia OR Gabon OR Gambia OR Ghana OR Guinea OR "Guinea-Bissau" OR Kenya OR Lesotho OR Liberia OR Madagascar OR Malawi OR Mali OR Mauritania OR Mauritius OR Mozambique OR Namibia OR Niger OR Nigeria OR Rwanda OR "Sao Tome and Principe" OR Senegal OR Seychelles OR "Sierra Leone" OR "South Africa" OR "South Sudan" OR Swaziland OR Togo OR Uganda OR ("United Republic of Tanzania" OR Tanzania) OR Zambia OR Zimbabwe) |
|  | **AND** |
| **Resistance OR Drug** | (TS=(("antimicrobial resistance" OR "antimicrobial resistant" OR AMR) OR ("Drug Resistance"[Mesh]) OR (Drug Resistance, Bacterial[Mesh]) OR ("multidrug resistance" OR "multidrug resistant" OR MDR) OR ("multiple antibiotic resistance" OR MAR[Title/Abstract]) OR (antibiotic AND resistan*) OR (resistan*) OR (carbapenem* OR carbapenems OR imipenem OR imipenems OR doripenem OR doripenems OR meropenem OR meropenems OR ertapenem OR ertapenems))) |
|  | **AND** |
| **Resistance OR Drug** | (TS=(("Gram-Negative Bacterial Infections"[Mesh]) OR "Healthcare-associated infection" OR "Cross-Infection"[Mesh] OR "community-associated infection" OR "Community-Acquired Infections"[Mesh] OR nosocomial OR (ESBL OR "ESBL isolates" OR "extended-spectrum β-lactamases" OR "extended-spectrum beta-lactamases") OR ("Enterobacteriaceae"[Mesh] OR "Enterobacteriaceae Infections"[Mesh]) OR ("carbapenem-resistant enterobacteriaceae" OR CRE OR "Carbapenem resistant enterobacteria*" OR carbapenem resistan* OR carbapenem) OR "Enterobacter" OR "Klebsiella" OR "Escherichia" OR (carbapenemase OR carbapenemases OR KPC OR GES OR IMP OR NDM OR OXA OR VIM))) |

| **Web of Science**  **Filters: From 1996-2019 and English** | |
| --- | --- |
| **Place** | (TS=(“Africa” OR Djibouti OR Egypt OR Libya OR Morocco OR Somalia OR Sudan OR Tunisia)) |
|  | **AND** |
| **Resistance OR Drug** | (TS=(("antimicrobial resistance" OR "antimicrobial resistant" OR AMR) OR ("Drug Resistance"[Mesh]) OR (Drug Resistance, Bacterial[Mesh]) OR ("multidrug resistance" OR "multidrug resistant" OR MDR) OR ("multiple antibiotic resistance" OR MAR[Title/Abstract]) OR (antibiotic AND resistan*) OR (resistan*) OR (carbapenem* OR carbapenems OR imipenem OR imipenems OR doripenem OR doripenems OR meropenem OR meropenems OR ertapenem OR ertapenems))) |
|  | **AND** |
| **Resistance OR Drug** | (TS=(("Gram-Negative Bacterial Infections"[Mesh]) OR "Healthcare-associated infection" OR "Cross-Infection"[Mesh] OR "community-associated infection" OR "Community-Acquired Infections"[Mesh] OR nosocomial OR (ESBL OR "ESBL isolates" OR "extended-spectrum β-lactamases" OR "extended-spectrum beta-lactamases") OR ("Enterobacteriaceae"[Mesh] OR "Enterobacteriaceae Infections"[Mesh]) OR ("carbapenem-resistant enterobacteriaceae" OR CRE OR "Carbapenem resistant enterobacteria*" OR carbapenem resistan* OR carbapenem) OR "Enterobacter" OR "Klebsiella" OR "Escherichia" OR (carbapenemase OR carbapenemases OR KPC OR GES OR IMP OR NDM OR OXA OR VIM))) |

| **Web of Science**  **Filters: From 1996-2019 and English** | |
| --- | --- |
| **Place** | (TS=("Africa" OR Algeria OR Angola OR Benin OR Botswana OR "Burkina Faso" OR Burundi OR Cameroon OR "Cabo Verde" OR ("Central African Republic" OR CAR) OR Chad OR Comoros OR Congo OR ("Cote d'Ivoire" OR "Ivory Coast") OR "Democratic Republic of the Congo" OR Djibouti OR Egypt OR "Equatorial Guinea" OR Eritrea OR Ethiopia OR Gabon OR Gambia OR Ghana OR Guinea OR "Guinea-Bissau" OR Kenya OR Lesotho OR Liberia OR Libya OR Madagascar OR Malawi OR Mali OR Mauritania OR Mauritius OR Morocco OR Mozambique OR Namibia OR Niger OR Nigeria OR Rwanda OR "Sao Tome and Principe" OR Senegal OR Seychelles OR "Sierra Leone" OR Somalia OR "South Africa" OR "South Sudan" OR Sudan OR Swaziland OR Togo OR Uganda OR ("United Republic of Tanzania" OR Tanzania) OR Tunisia Or Zambia OR Zimbabwe) |
|  | **AND** |
| **Resistance OR Drug** | (TS=("antimicrobial resistance" OR "antimicrobial resistant" OR AMR) OR ("Drug Resistance"[Mesh]) OR (Drug Resistance, Bacterial[Mesh]) OR ("multidrug resistance" OR "multidrug resistant" OR MDR) OR ("multiple antibiotic resistance" OR MAR[Title/Abstract]) OR (antibiotic AND resistan*) OR (resistan*) OR Colistin OR polymyxin OR "polymyxin E" OR "polymyxin B")) |
|  | **AND** |
| **Resistance OR Drug** | (TS=("Gram-Negative Bacterial Infections"[Mesh]) OR "Healthcare-associated infection" OR "Cross-Infection"[Mesh] OR "community-associated infection" OR "Community-Acquired Infections"[Mesh] OR nosocomial OR (ESBL OR "ESBL isolates" OR "extended-spectrum β-lactamases" OR "extended-spectrum beta-lactamases") OR ("Enterobacteriaceae"[Mesh] OR "Enterobacteriaceae Infections"[Mesh]) OR (“polymyxin resistant Enterobacteriaceae”) OR (“colistin resistant Enterobacteriaceae”) OR “Enterobacter" OR "Klebsiella" OR "Escherichia" OR (mcr OR "mcr-1" OR “mcr-2” OR "mcr-3"))) |

| **Embase**  **Filters: 2017–2019 and Human**  **Global Health**  **Filters: 2017–2019, English, and Human** | |
| --- | --- |
| **Place** | 1. (Africa or Algeria or Angola or Benin or Botswana or "Burkina Faso" or Burundi or Cameroon or "Cabo Verde" or ("Central African Republic" or CAR) or Chad or Comoros or Congo or ("Cote d'Ivoire" or "Ivory Coast") or "Democratic Republic of the Congo" or "Equatorial Guinea" or Eritrea or Ethiopia or Gabon or Gambia or Ghana or Guinea or "Guinea-Bisseau" or Kenya or Lesotho or Liberia or Madagascar or Malawi or Mali or Mauritania or Mauritius or Mozambique or Nambia or Niger or Nigeria or Rwanda or "Sao Tome and Principe" or Senegal or Seychelles or "Sierra Leone" or "South Africa" or "South Sudan" or Swaziland or Togo or Uganda or ("United Republic of Tanzania" or Tanzania) or Zambia or Zimbabwe or Morocco or Tunisia or Libya or Egypt or Somalia or ("South Sudan" or Sudan)).ab,ot,sh,ti. |
| **Resistance OR Drug** | 1. ("antimicrobial resistance" or "antimicrobial resistant" or AMR or "Drug Resistance" or bacterial drug resistance or ("Multidrug Resistance" or "multidrug resistant" or MDR) or ("multiple antibiotic resistance" or MAR) or (("Antibiotic Resistance" or antibiotic) and resistan*) or resistan* or (carbapenem* or carbapenems or "Carbapenem Derivative" or imipenem or imipenems or doripenem or doripenems or meropenem or meropenems or ertapenem or ertapenems)).ab,ot,sh,ti. |
| **Resistance OR Drug** | 1. ("gram-negative bacterial infections" or "Gram Negative Infection" or "Healthcare Associated Infection" or "Hospital Infection" or "Cross-Infection" or "community-associated infection" or "Community Acquired Infection" or "community-acquired infections" or nosocomial or (ESBL or "ESBL isolates" or "extended-spectrum β-lactamases" or "extended-spectrum beta-lactamases") or ("Enterobacteriaceae" or "Enterobacteriaceae Infection" or "enterobacteriaceae infections") or ("Carbapenem Resistant Enterobacteriaceae" or CRE or "Carbapenem resistant enterobacteria*" or carbapenem resistan* or carbapenem) or "Enterobacter" or "Klebsiella" or "Klebsiella Infection" or "Escherichia" or (carbapenemase or carbapenemases or KPC or GES or IMP or NDM or OXA or VIM)).ab,ot,sh,ti. |
|  | 4. 1 and 2 and 3 |
|  | 5. limit 4 to (human and yr="2017-2019") |
|  | 6. limit 5 to (english language and yr="2017-2019") |

| **Embase**  **Filters: 1996–2019 and Human**  **Global Health**  **Filters: 1996–2019, English, and Human** | |
| --- | --- |
| **Place** | 1. (Africa or Djibouti or Egypt or Libya or Morocco or Somalia or Sudan or Tunisia).ab,ot,sh,ti. |
| **Resistance OR Drug** | 1. ("antimicrobial resistance" or "antimicrobial resistant" or AMR or "Drug Resistance" or bacterial drug resistance or ("Multidrug Resistance" or "multidrug resistant" or MDR) or ("multiple antibiotic resistance" or MAR) or (("Antibiotic Resistance" or antibiotic) and resistan*) or resistan* or (carbapenem* or carbapenems or "Carbapenem Derivative" or imipenem or imipenems or doripenem or doripenems or meropenem or meropenems or ertapenem or ertapenems)).ab,ot,sh,ti. |
| **Resistance OR Drug** | 1. ("gram-negative bacterial infections" or "Gram Negative Infection" or "Healthcare Associated Infection" or "Hospital Infection" or "Cross-Infection" or "community-associated infection" or "Community Acquired Infection" or "community-acquired infections" or nosocomial or (ESBL or "ESBL isolates" or "extended-spectrum β-lactamases" or "extended-spectrum beta-lactamases") or ("Enterobacteriaceae" or "Enterobacteriaceae Infection" or "enterobacteriaceae infections") or ("Carbapenem Resistant Enterobacteriaceae" or CRE or "Carbapenem resistant enterobacteria*" or carbapenem resistan* or carbapenem) or "Enterobacter" or "Klebsiella" or "Klebsiella Infection" or "Escherichia" or (carbapenemase or carbapenemases or KPC or GES or IMP or NDM or OXA or VIM)).ab,ot,sh,ti. |
|  | 4. 1 and 2 and 3 |
|  | 5. limit 4 to (human and yr="1996-2019") |
|  | 6. limit 5 to (english language and yr="1996-2019") |

| **Embase**  **Filters: 1996–2019 and Human**  **Global Health**  **Filters: 1996–2019, English, and Human** | |
| --- | --- |
| **Place** | 1. (Africa or Algeria or Angola or Benin or Botswana or "Burkina Faso" or Burundi or Cameroon or "Cabo Verde" or ("Central African Republic" or CAR) or Chad or Comoros or Congo or ("Cote d'Ivoire" or "Ivory Coast") or "Democratic Republic of the Congo" or Djibouti or Egypt or "Equatorial Guinea" or Eritrea or Ethiopia or Gabon or Gambia or Ghana or Guinea or "Guinea-Bisseau" or Kenya or Lesotho or Liberia or Libya or Madagascar or Malawi or Mali or Mauritania or Mauritius or Morocco or Mozambique or Nambia or Niger or Nigeria or Rwanda or "Sao Tome and Principe" or Senegal or Seychelles or "Sierra Leone" or Somalia or "South Africa" or "South Sudan" or Sudan or Swaziland or Togo or Uganda or ("United Republic of Tanzania" or Tanzania) or Tunisia or Zambia or Zimbabwe).ab,ot,sh,ti. |
| **Resistance OR Drug** | 1. ("antimicrobial resistance" or "antimicrobial resistant" or AMR or "Drug Resistance" or bacterial drug resistance or ("Multidrug Resistance" or "multidrug resistant" or MDR) or ("multiple antibiotic resistance" or MAR) or (("Antibiotic Resistance" or antibiotic) and resistan*) or resistan* or colistin or polymyxin or "polymyxin e" or "polymyxin b").ab,ot,sh,ti. |
| **Resistance OR Drug** | 1. ("gram-negative bacterial infections" or "Gram Negative Infection" or "Healthcare Associated Infection" or "Hospital Infection" or "Cross-Infection" or "community-associated infection" or "Community Acquired Infection" or "community-acquired infections" or nosocomial or (ESBL or "ESBL isolates" or "extended-spectrum β-lactamases" or "extended-spectrum beta-lactamases") or ("Enterobacteriaceae" or "Enterobacteriaceae Infection" or "enterobacteriaceae infections") or "colistin resistant enterobacteria*" or "polymyxin resistant enterobacteria*" or "Enterobacter" or "Klebsiella" or "Klebsiella Infection" or "Escherichia" or (mcr or "mcr-1" or "mcr-2" or "mcr-3")).ab,ot,sh,ti. |
|  | 4. 1 and 2 and 3 |
|  | 5. limit 4 to (human and yr="1996-2019") |
|  | 6. limit 5 to (english language and yr="1996-2019") |
